# Conceptual model for the health technology assessment of current and novel interventions in rheumatoid arthritis

**DOI:** 10.1371/journal.pone.0205013

**Published:** 2018-10-05

**Authors:** Evo Alemao, Maiwenn J. Al, Annelies A. Boonen, Matthew D. Stevenson, Suzanne M. M. Verstappen, Kaleb Michaud, Michael E. Weinblatt, Maureen P. M. H. Rutten-van Mölken

**Affiliations:** 1 Worldwide Health Economics and Outcomes Research, Bristol-Myers Squibb (BMS), Lawrence, New Jersey, United States of America; 2 Erasmus University Rotterdam, Rotterdam, The Netherlands; 3 Erasmus School of Health Policy and Management, Erasmus University Rotterdam, Rotterdam, The Netherlands; 4 Department of Internal Medicine, Division of Rheumatology, Maastricht University Medical Centre, Maastricht University, Maastricht, The Netherlands; 5 School of Health and Related Research, University of Sheffield, Sheffield, United Kingdom; 6 Arthritis Research UK Centre for Epidemiology, Centre for Musculoskeletal Research, Faculty of Biology, Medicine and Health, University of Manchester, Manchester Academic Health Science Centre, Manchester, United Kingdom; 7 Department of Rheumatology and Immunology, University of Nebraska Medical Center, Omaha, New England, United States of America; 8 Division of Rheumatology, Immunology and Allergy, Brigham and Women’s Hospital, Harvard University, Boston, Massachusetts, United States of America; 9 Institute for Medical Technology Assessment (iMTA), Erasmus University Rotterdam, Rotterdam, The Netherlands; VU University Medical Center, NETHERLANDS

## Abstract

The objective of this study was to evaluate current approaches to economic modeling in rheumatoid arthritis (RA) and propose a new conceptual model for evaluation of the cost-effectiveness of RA interventions. We followed recommendations from the International Society of Pharmacoeconomics and Outcomes Research-Society of Medical Decision Making (ISPOR-SMDM) Modeling Good Research Practices Task Force-2. The process involved scoping the decision problem by a working group and drafting a preliminary cost-effectiveness model framework. A systematic literature review (SLR) of existing decision-analytic models was performed and analysis of an RA registry was conducted to inform the structure of the draft conceptual model. Finally, an expert panel was convened to seek input on the draft conceptual model. The proposed conceptual model consists of three separate modules: 1) patient characteristic module, 2) treatment module, and 3) outcome module. Consistent with the scope, the conceptual model proposed six changes to current economic models in RA. These changes proposed are to: 1) use composite measures of disease activity to evaluate treatment response as well as disease progression (at least two measures should be considered, one as the base case and one as a sensitivity analysis); 2) conduct utility mapping based on disease activity measures; 3) incorporate subgroups based on guideline-recommended prognostic factors; 4) integrate realistic treatment patterns based on clinical practice/registry datasets; 5) assimilate outcomes that are not joint related (extra-articular outcomes); and 6) assess mortality based on disease activity. We proposed a conceptual model that incorporates the current understanding of clinical and real-world evidence in RA, as well as of existing modeling assumptions. The proposed model framework was reviewed with experts and could serve as a foundation for developing future cost-effectiveness models in RA.

## Introduction

Rheumatoid arthritis (RA) is a progressive disease characterized by inflammation of synovial tissue with symmetric involvement of peripheral joints of the hand, feet, and wrists[1,2]. The prevalence of RA ranges from 0.4% to 1.3% [3]. RA not only contributes to reduced survival, health related quality of life (HRQOL), activities of daily living and work productivity, but is also associated with higher health resource utilization and costs compared to general population and osteoarthritis patients [4–7]. Most RA-related direct costs are associated with biologic disease modifying anti-rheumatic drugs (bDMARDs), which have improved outcomes in RA patients [8–11]. Since the introduction of these agents, our knowledge of RA as a disease has greatly increased and new therapies as well as combination therapies (of different bDMARDs or of bDMARDs in combination with synthetic (sc)DMARDs) targeting multiple immune pathways are being developed [12,13]. The development of novel interventions is accompanied by the introduction of bioequivalents or biosimilars of existing bDMARDs. In an environment featuring multiple therapeutic options to manage RA patients on one side and constrained health resources on the other, cost-effectiveness models that enable more precise estimations of cost and benefits could reduce the risk of inefficient resource allocation.

The framework for cost-effectiveness models for treatments in RA has evolved since first published in early 2000s, with the introduction of bDMARDs [14,15]. The current modeling approach has served to establish economic benefits of bDMARDs in most countries, in moderate to severe RA patients with inadequately respond to methotrexate [16,17]. In our opinion previously, published models have potential room for improvement in six areas. First, current models base treatment response on composite measures of disease activity such as European League Against Rheumatism (EULAR) response [18], American College of Rheumatology (ACR) response [19], and Disease Activity Scores in 28 joints C-reactive protein (DAS28-CRP) [20]. These disease activity measures are not aligned to guideline-recommended target measures of remission and hence cannot evaluate the cost-effectiveness of policies designed to implement treatment guideline-based targets [21,22]. In addition, these measures are biased (favorably) to certain therapeutic interventions that disproportionately impact individual components of the composite measure for example CRP with interleukin-6 (IL-6) inhibitors; this is discussed further in results section under new conceptual model [23].

Second, disease progression in these models is based on physical functioning measured by the Health Assessment Questionnaire (HAQ) [24]. HAQ changes are related to inflammatory disease activity in early RA and predominately to structural damage in longstanding RA. Thus more rapid decline in HAQ on treatment is observed in patients with RA of recent onset, compared to those with established RA [25]. The greater reduction in HAQ observed with treatments in patients with early versus established RA highlights the ceiling effect of HAQ and thus may be insensitive to beneficial treatment effects.

Third, contemporary models derive utility scores from the HAQ, based on mapping algorithms. Nonlinear models are now recommended, and overall mapping of HAQ to European Quality of life 5 dimension (EQ-5D) [26] has been improved by including disease activity and pain in these models [27,28]. However, no study (to our knowledge) has evaluated the impact of other dimensions of RA or of different composite measures on utility scores.

Fourth, certain baseline characteristics, such as age, gender, and HAQ score, are accounted for in current models. However, most of these models do not report incremental cost-effectiveness ratios (ICERs) according to important subgroups. Recent studies have evaluated ICER within a limited number of RA subgroups [29,30].

Fifth, current modeling approaches focus on joint-related outcomes in RA, largely at the expense of extra-articular manifestations. Extra-articular manifestations occur in 18% to 41% of patients with RA [31–35]. A growing body of evidence—mainly derived from observational databases and registries—suggests that specific RA therapies, including methotrexate and bDMARDs, may reduce the risk of extra-articular cardiovascular disease [CVD] manifestations with RA [36,37].

Sixth and finally, long-term treatment discontinuation in current models is based on real-world registry data, and these models allow for patients to cycle through tumor necrosis factor inhibitor (TNF-i) via limited sequential use of bDMARDs [28,38]. However, these same models do not allow for data in which health providers escalate doses or re-initiate bDMARDs once treatment has been discontinued or stopped if the patient experiences a flare [39]. These factors may result in underestimating both increasing therapeutic benefits and costs. Although contemporary RA therapy is moving toward lowering the dose of the bDMARDs in patients once they have attained a predefined target disease activity state, such dose de-escalation is not incorporated in previously reported models [40–43].

Taken together, these factors point to unmet needs related to pharmacoeconomic modeling in RA. Consideration of these aspects in future economic modeling of RA treatments could enable evaluation of costs and benefits of therapies in manner that reflects prevailing clinical realities with the aim of producing more accurate cost-effectiveness estimates. The objective of this analysis was to review current economic models in RA and propose a revised conceptual model framework.

## Methods

**Fig 1:**
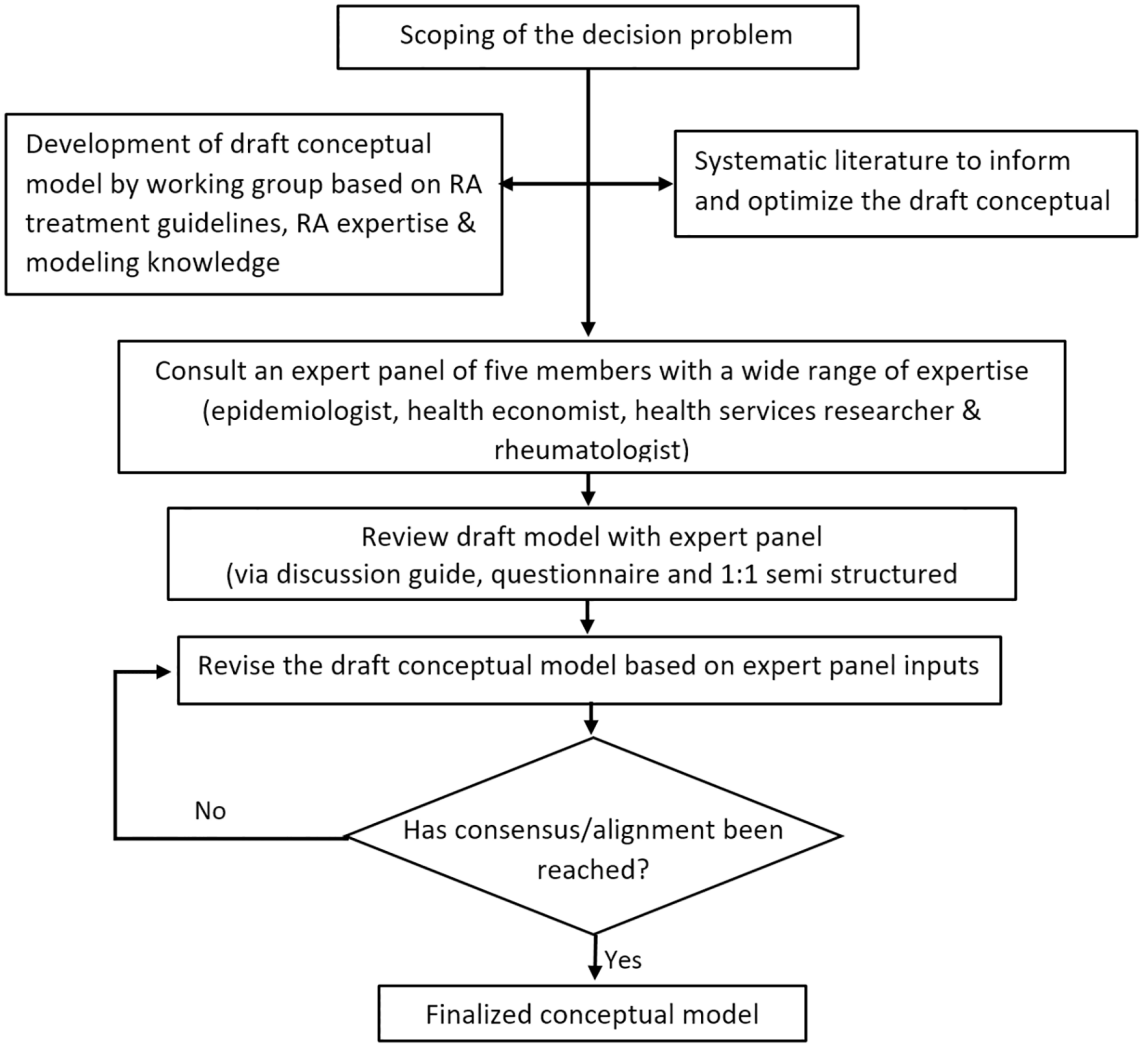
Schematic overview of the development process of the conceptual model.

In developing the conceptual model, the recommendations outlined by the International Society of Pharamacoeconomic and Outcomes Research-Society of Medical Decision Making (ISPOR-SMDM) Modeling Good Research Practices Task Force-2 were followed [44]. As depicted in Fig 1, the process involved scoping out the decision problem by a working group and drafting a preliminary cost effectiveness model framework. A systematic literature review (SLR) of existing decision-analytic models was performed and analysis of a RA registry was conducted to inform the structure of the draft conceptual model. Finally, an expert panel was convened to seek input on the draft conceptual model.

### Scoping

The knowledge gaps in current models as explained in the introduction were elucidated in a three-member (EA, MA, MR) working group. The working group focused on various aspects of the model, such as 1) measures to access treatment responses/treatment targets, 2) measure to assess RA disease progression, 3) utility mapping, 4) RA subgroups, 5) treatment patterns (e.g. dose escalation, dose de-escalation) 6) extra-articular outcomes, and mortality. Based on these aspects the working group put together a list of revisions of existing models (S1 Appendix) and drafted a conceptual model (S2 Appendix). The draft conceptual model was based on the working group analyses of a RA registry to explore treatment targets and extra-articular manifestation of RA, and RA subgroups, which have been published elsewhere [45–48]. In addition, the working group relied on literature and knowledge of clinical guidelines to inform model development.

### SLR

A review of existing decision-analytic models on the cost-effectiveness of RA therapies published in English since 2006 was conducted as part of the scoping process. The search strategy is depicted in S3 Appendix. Primary searches were conducted in Medline, EMBASE, and EconLit simultaneously using Ovid based on the search strategy outlined. In addition to the SLR, recent publication on methodologies of economic modeling in RA was also reviewed [49]. To supplement the database search, a manual search of previous health technology assessment (HTA) reports was conducted on the UK National Institute for Health and Clinical Excellence website (https://www.nice.org.uk/guidance/ta375/history [last accessed Nov 2017]). The primary objective of the SLR was to identify published economic evaluations of bDMARDs in RA. The SLR focused on model structure, short-term treatment targets/responses, RA disease progression (long-term response when initial treatment is successful), utility mapping, patient subgroups (with characteristics that could be treatment effect modifiers), treatment aspects (switching, dose escalation, de-escalation), time horizon, and mortality associated with RA.

### Analysis to inform conceptual model

To inform disease progression and utility mapping in the conceptual model, the working group conducted a retrospective analysis of a RA registry. A longitudinal sequential registry of primarily established RA patients was used for this analysis. In this registry, disease activity was measured annually during rheumatology visits using multiple composite functional measures [50]. These included the DAS28-CRP, Simplified Disease Activity Index (SDAI) and Clinical Disease Activity Index (CDAI) [51]. The generic HRQOL index EQ-5D was evaluated every 6 months via both mailed questionnaires and in-person interview (during annual visit). The progression of RA using various composite measures as well as changes in these disease activity over time was evaluated using general linear models. Mapping algorithms based on DAS28-CRP, SDAI, and CDAI were compared to the physical functioning (HAQ)-based mapping algorithm. Fixed-effects models were used to estimate the best predictors of EQ-5D, because within-patient variability over time is more important than between patient variability in economic models [52].

### Expert panel

An expert panel comprising two rheumatologist (AB, MW), one health economist (MS), and two epidemiologists/health services researchers (KM, SV) was convened to provide input to the conceptual model. The draft conceptual model was presented to each expert in a multistep approach. In the first step, a member of the working group (EA) shared the discussion guide developed by the working group with the expert panel members. The discussion guide contained an overview and limitations of current modeling approaches in RA as well as the proposed conceptual model structure. It also included a brief questionnaire that focused on the proposed modifications to the cost-effectiveness model. In the second step, opinions from all experts of the panel were gathered via individual interviews. The third step involved updating the draft conceptual model and collating all responses to questions and comments. The revised document was shared with all experts for additional inputs. Additional updates were then incorporated, and the conceptual model was sent back to the panel for a final opportunity to provide suggestions.

## Results

### Scoping

The decision problem that the conceptual model would address was defined as identifying cost-effective drug interventions for moderate-to-severe RA that result in the most health benefits for the overall RA population as well as for specific subgroups (such as those with poor prognostic factors). This includes current and novel interventions that are being developed and may be introduced in clinical practice in the future as monotherapy or combination therapies.

### SLR

A total of 32 economic evaluation studies were identified by the initial SLR, 5 of which were review articles. The remaining 27 manuscripts evaluated are summarized in S3 Appendix Table 3 [29,38,49–77]. The primary model structures were cohort based or individual patient simulations, which included discrete event simulations and individual patient Markov models. More recent published models tended to be primarily individual patient simulations.

**Fig 2:**
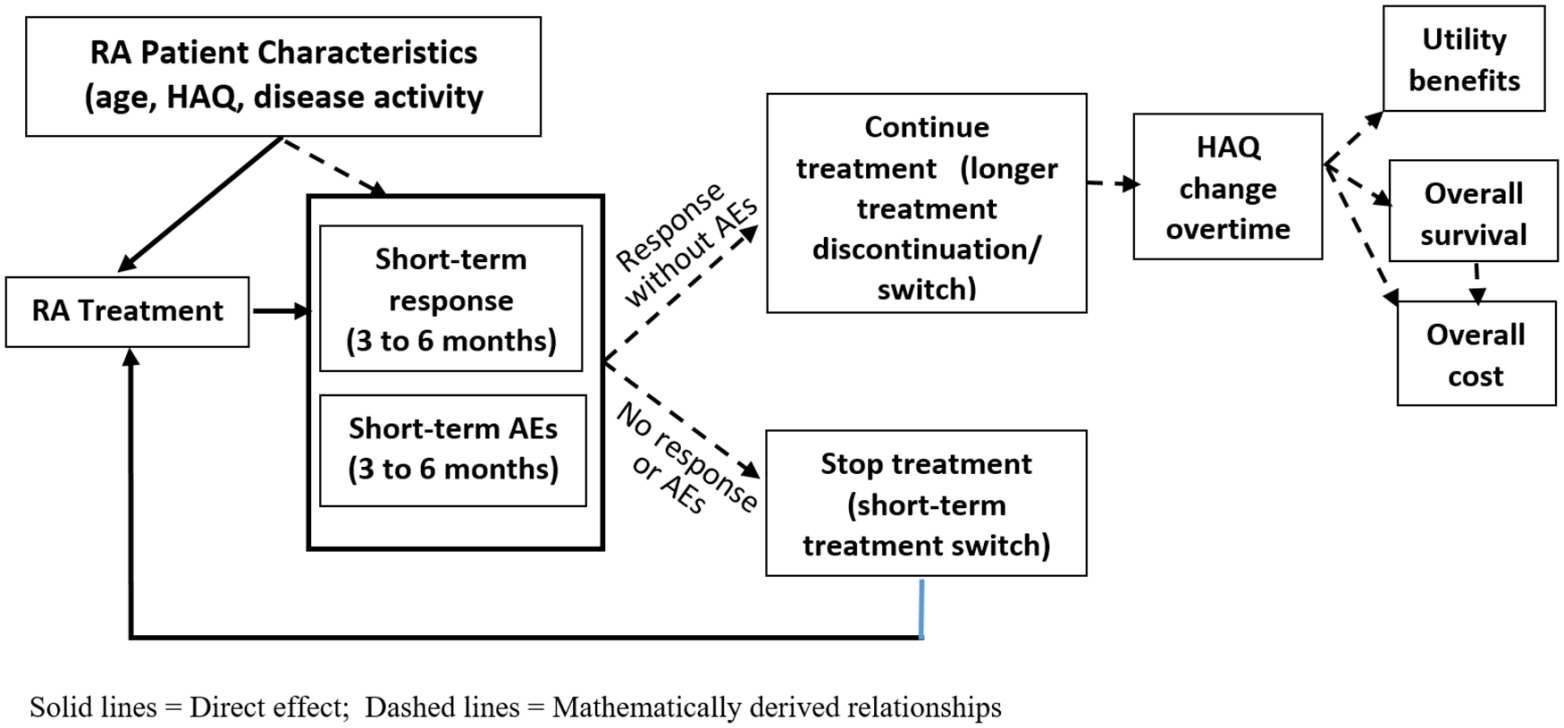
RA economic model influence diagram for structural relationship.

The assumed relationship between different model variables is summarized in the influence diagram represented in Fig 2. Each solid arrow represents a direct effect of one variable on the other, while the dashed lines represent the mathematically derived structural relationships. In general, these models evaluate short-term (3 to 6 months) treatment effects based on clinical trial efficacy. Based on the short-term efficacy and probability of adverse events (AEs), a decision rule was included in the model for a patient to continue treatment or not. If treatment is continued, then disease progression is estimated based on HAQ change over time. In recent models HAQ change over time is based on mixture models, while earlier models used linear progression (0 per annum for bDMARDs and 0.03 to 0.045 per annum for cDMARDs) [29,64]. HAQ scores are then mapped to HRQOL, mortality rates and resource use, using mapping algorithms. The long-term treatment duration in the majority of the simulation models is based on real-world registry data, extrapolated using survival models with time to treatment discontinuation as outcomes. The endpoint driving cost-effectiveness models in RA is primarily physical functioning, whereas other endpoints such as radiographic progression are rarely used [76].

### New conceptual model

**Fig 3:**
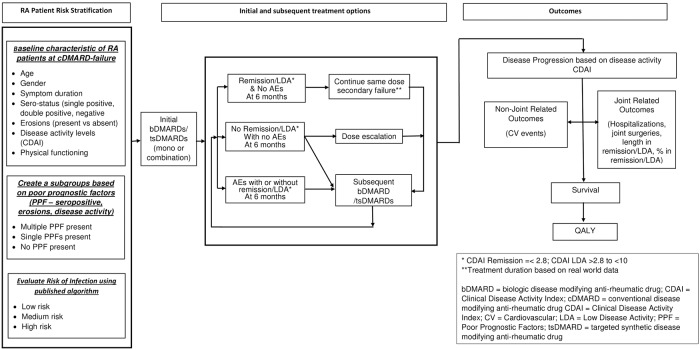
Draft conceptual model to evaluate cost effectiveness in RA.

The conceptual model drafted by the working group is represented in Fig 3. The proposed conceptual model is an individual patient simulation model with a lifetime horizon proposed to capture short-term and long-term benefits and cost of interventions. Outcomes are defined as quality-adjusted life years (QALYs) and life-years gained (LYG). This model is intended for HTA and is based on the payer’s perspective and has three distinct modules: 1) *patient characteristic module*, 2) *treatment module* and 3) *outcome module*. This framework enables addressing issues of treatment responses, RA subgroups, real world treatment patterns and extra-articular manifestation of RA mentioned in the introduction. This proposed conceptual model should be seen as “aspirational” because not all data elements required to populate the model are available (at the time of writing) but are likely to become available in the future. The ISPOR-SMDM Modeling Good Research Practices Task Force-2 stresses that conceptual models should not be driven solely by the presence or absence of clinical data [44].

To improve the clinical relevance of the economic models in RA, it is important to align treatment responses to guideline-recommended targets, which are based on composite measures of disease activity [21,22]. The working group proposed the expert panel to consider CDAI as a measure of treatment response in the conceptual model (change 1a). This was primarily based on observed associations between treatment targets and outcomes of physical functioning (HAQ), HRQOL (i.e. EQ-5D), and health resource use. A greater improvement was observed in these outcomes among patients attaining (vs. not attaining) a CDAI based target, compared to SDAI and DAS28-CRP based targets [47]. In addition, CDAI has acceptable psychometric properties, including validity and sensitivity to change [78–81]. CDAI remission does not include levels of CRP or erythrocyte sedimentation rate (ESR) which are primarily impacted by therapies such as IL-6 and janus kinase inhibitors. Thus, the new conceptual model could include a CDAI score of <2.8 (remission) or ≤10 (LDA) as a definition for responder for treatment continuation. In terms of disease progression, the working group proposed CDAI change over time (change 1b). This was based on analysis of changes in CDAI, SDAI and DAS28-CRP in a cohort of patients with mostly established RA [50]. Results of these analyses indicate that response to change over time is dependent on disease duration and measurement of disease activity under consideration (S4 Appendix).

Finally, the mapping exercise of disease activity measures and physical functioning to EQ-5D indicates that mapping models predicated on CDAI and Routine Assessment of Patient Index Data-3 (RAPID-3) measures have the best fit according to *r*^2^ and root mean square error values (Table 1). RAPID-3 is an index of physical function, patient pain, and patient global, and as such a PRO index of disease activity (or impact). Based on these findings, the working group proposed to the expert panel that the conceptual framework should include utilities based on disease activity measure (CDAI) and RAPID-3 (change 2).

**Table 1:** Fixed effects regression models for EQ5D.

Models	R-Square	Root MSE	F- value
Patient global, Patient pain scale RADAI Joint Score	0.70	0.09	14.2
RAPID3, RADAI Joint Score	0.72	0.09	14.3
RAPID3	0.71	0.09	13.8
**RAPID3, CDAI**	**0.75**	**0.09**	**7.4**
mHAQ	0.68	0.10	15.4
mHAQ, RADAI Joint Score	0.70	0.09	16.7
mHAQ, CDAI	0.71	0.09	7.8
mHAQ, mHAQ square	0.68	0.10	15.4
mHAQ, pain	0.70	0.09	14.4
mdHAQ, RADAI Joint Score	0.70	0.09	17.0
mdHAQ	0.68	0.10	15.8
**Models with baseline co-variates of age, duration, CRP and serostatus**			
Patient global, Patient pain scale RADAI Joint Score	0.74	0.09	7.5
RAPID3, RADAI Joint Score	0.73	0.09	15.1
RAPID3	0.71	0.09	14.0
**RAPID3, CDAI**	**0.75**	**0.09**	**7.4**
mHAQ	0.68	0.10	15.4
mHAQ, RADAI Joint Score	0.69	0.09	16.7
mHAQ, CDAI	0.71	0.09	7.8
mHAQ, mHAQ square	0.68	0.10	15.4
mHAQ, pain	0.70	0.09	14.5
mdHAQ, RADAI Joint Score	0.70	0.09	14.6
mdHAQ	0.68	0.10	15.7

Based on current evidence, the conceptual model accommodates subgroups with a high risk of disease progression such as those with multiple prognostic factors (change 3). Additional subgroups that the working group considered important for inclusion were patients with susceptible to infections. The *patient characteristic module* accounts for patient characteristics when entering the model and at subsequent time points. This module enables risk stratification of RA patients based on prognostic factors. Some commonly reported prognostic factors for a more rapid and aggressive disease are double seropositivity for anti-cyclic citrullinated peptide antibody (ACPA) and rheumatoid factor (RF), as well as erosions, disease activity and measures of inflammation (CRP/ESR) [81–85]. There is evidence that certain prognostic factors can be considered as treatment effect modifiers [86–88]. Subgroups based on patients’ risks of infections were considered, RA patients with high disease activity also have increased risk of infections as well as CVD [89,90]. In addition, evidence indicates that glucocorticoids and certain DMARDs increase the risk of infection in RA patients [91,92] and because prevalent RA patients tend to be elderly and thus at increased risk for infections.

*The treatment module* accommodates all treatment changes (change 4), in patients who do not attain remission or low disease activity (LDA) or patients who experience AEs within 3 to 6 months (or secondary failure) after treatment initiation. In addition, the proposed conceptual model allows for flexibility in dose escalation. Data from observational studies have shown that some patients require upward dose adjustments, reduced dose interval for bDMARDS, or addition of glucocorticoids and/or nonsteroidal anti-inflammatory drugs (glucocorticoids/NSAIDs) to some bDMARDs in order to achieve or maintain a clinical response [93,94]. Upward dose adjustments are associated with increased medication costs and potentially adverse reactions. Dose escalation is not in the summary of the product characteristics of any of the current approved DMARDs.

*The outcome module* incorporates disease progression and its impact on both joint and extra-articular outcomes. The conceptual model accommodates extra-articular disease outcomes, principally CVD events (change 5). These events were considered by the working group primarily based on available epidemiologic data, as well as on the treatment effects and the cost implications of these outcomes. The working group proposed incorporation of RA-specific mortality risk based on disease activity in the economic model once more data becomes available (change 6).

### Expert panel

Members of the expert panel debated the draft model structure, challenging the level of evidence to support several proposed changes. Nevertheless, a majority of the panel agreed that the model should enable subgroup analysis by prognostic factors, and also investigate the need to accommodate increased risk of infection (change 3). The experts agreed on QALYs should be the main outcome and, mortality based on RA disease activity (change 6). In addition, there was agreement on further exploring the impact of including extra-articular manifestations on ICERs (change 5).

The expert panel also acknowledged the advantage of having a disease activity measure for both treatment response and disease progression (change 1). Questions were raised on CDAI data availability from historic phase 3 programs and concerns were mentioned about the subjective elements of CDAI, such as estimation of tender joint counts, patient and physician global health, which are unweighted and can make the measure less reliable. At the same time, the members of the expert panel acknowledged that this perceived limitation might also apply to other currently available composite measures. The least agreement among experts was on the proposed mapping of only disease activity (change 2) to HRQOL utilities (i.e. EQ-5D). Recommendation was to explore the use of mixed logit models, based on disease activity and HAQ with other dimensions of RA such as pain, fatigue. Strengths and limitations of the recommended changes, along with expert inputs and level of agreement among experts concerning the proposed changes, are summarized in Table 2.

**Table 2:** Summary of pros and cons of proposed changes, expert input and agreement.

Changes proposed	Pros and Cons	Expert Inputs	Expert Agreement*
Model Structure	Pros: aligned with clinical practice & guidelines; allows to captures patient subgroups, treatment heterogeneity, non-joint outcomes; Cons: increase in complexity; data availability	Ideal, however data may not be available to populate model Include cDMARD-naïve and cDMARD inadequate responders Changes may not materially impact ICER The time involved in incorporating the changes might not be worth the extra accuracy	3 of 5
Minimum of two disease activity measures for treatment response and disease progression	Pros: Aligns to treatment guidelines; less biased estimates (vs. single measure) Con: Data availability;	Data availability might be an issue	4 of 5
Disease activity based mapping of utilities	Pros: Addresses the limitation of HAQ changes; Allows the model to be based entirely on disease activity; could lead to further improvements in mapping of utilities Cons: Data availability	HAQ would still be an unbiased estimator of disease progression Reasons for HAQ was its association to cost in RA Would not recommend RAPID3 by itself as it based entirely on patient report. Good to see that we are combining disease activity and RAPID3	3 of 5
Incorporation of subgroups	Pros: Allows for specific and targeted HTA evaluations Cons: No general agreement that the prognostic factors are well established in RA; data availability	Double sero-positives are at a higher risk of progressing (vs. single positive) Patients who have erosive disease at baseline are high risk of progression Additional subgroups could include elderly i.e. age >65 yrs (as they are increased risk of infections), CV and other RA extra-articular manifestations These are not just baseline factors	5 of 5
Real world treatment patterns:	Pros: Allows for realistic estimates of cost and clinical benefits of standard of care Cons: data availability;	Generalizability of real world data vs. trials (where efficacy was gained) No controlled studies have examined switching therapy in patients who are well controlled GPs behavior cannot be clearly defined and consistent for dose reduction	4 of 5
Incorporating extra-articular manifestations of RA:	Pros: Allows for improved estimation of benefit and cost of interventions Cons: data availability;	CV and lung disease should be considered Important if treatment would differentially impact extra-articular manifestations The strength of this evidence, particularly with respect to changes in markers and changes in hard outcomes is limited	5 of 5
Mortality Associated with RA	Pros: allows for disease activity be the driver of benefits Cons: potential for overestimation of survival; data availability	No comments	5 of 5

*Agreement in principal that these need to be evaluated in future economic models;

IR–inadequate response; ICER = Incremental cost effectiveness ratio

**Fig 4:**
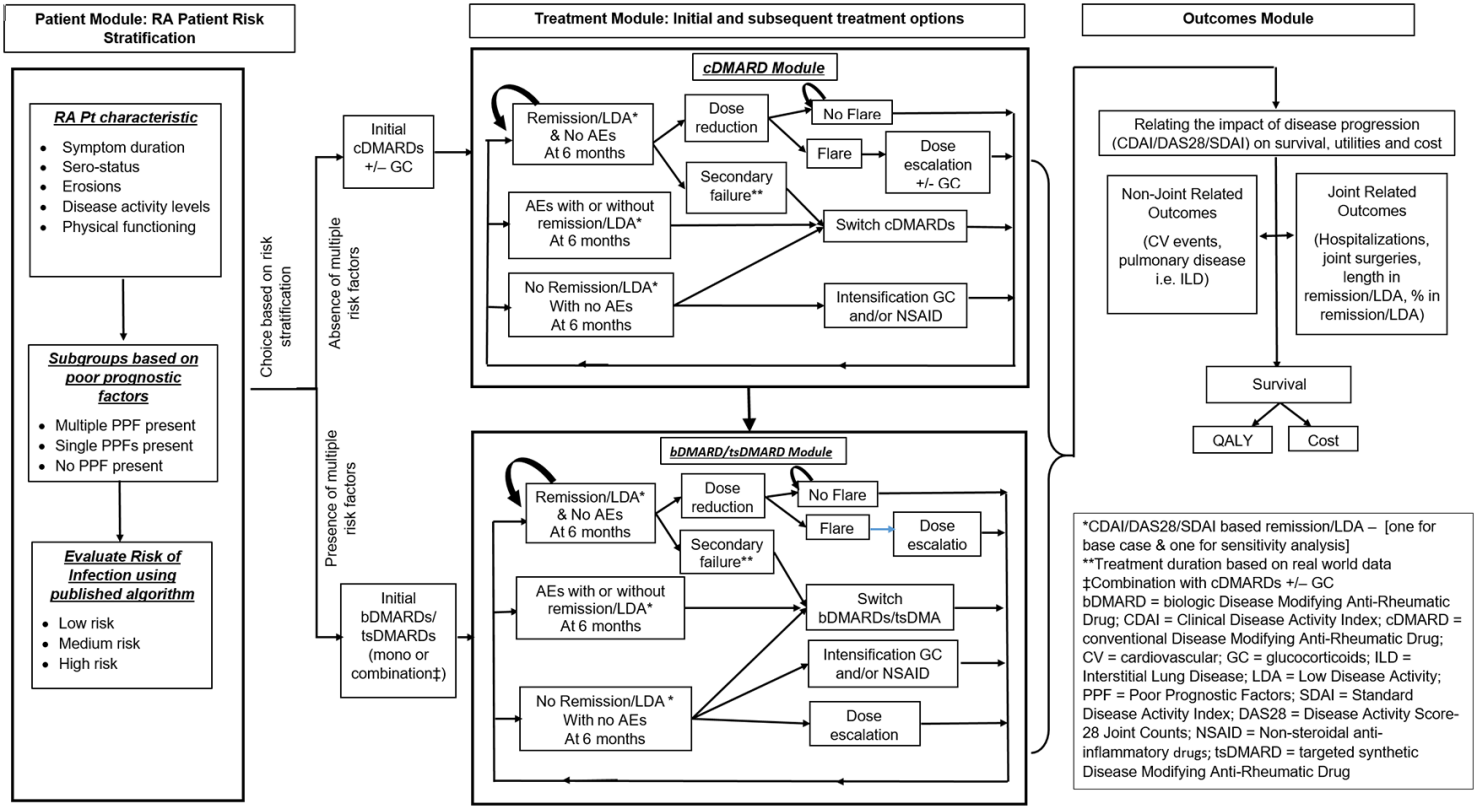
Updated conceptual model to evaluate cost effectiveness in RA.

After expert panel inputs had been incorporated, the draft conceptual model was further modified and these further changes are reflected in the updated conceptual model (Fig 4). Because there is no clinical criterion or reference standard disease activity measure, the conceptual framework was revised to incorporate at least two disease activity measures: one as a “base case” and the other for sensitivity analyses concerning treatment effect as well as disease progression (change 1). For example CDAI or SDAI or other disease activity measures could be used as the base case and DAS28-CRP for a sensitivity analysis. The updated conceptual model also includes, in the same framework, patients who have not been exposed to csDMARDs or who have not responded adequately to them. According to input from the expert panel, the conceptual model included treatment intensification (glucocorticoids and/or NSAIDs) before the patient received bDMARD switch and also dose de-escalation in patients attaining remission (change 4). The final update based on expert input was the inclusion of pulmonary extra-articular manifestation in addition to CV extra-articular effects of RA (change 5).

## Discussion

This study used a well-established methodology to propose a conceptual framework for developing future models in RA to evaluate the cost effectiveness of therapies [44]. The current cost effectiveness modeling framework in RA was introduced with the advent of TNF-i. Since then our knowledge of RA disease mechanism, impact on joints as well as on other organ systems has greatly increased. In addition, maturation of existing electronic medical records, claims datasets and registries enable us to better understand RA treatment patterns. Thus, the proposal of an updated conceptual model that incorporates these understandings may be timely. In proposing the conceptual model we leveraged the earlier modeling approaches as certain aspects are well established.

Overall, the proposed conceptual model reflects on 6 preselected areas of modelling cost-effectiveness of drug treatment in moderate to severe RA in the 21th century. The major changes that this conceptual model proposes are 1) use of at least two composite measures of disease activity, with one used in sensitivity analyses, to evaluate both treatment response as well as disease progression; 2) utility mapping based on disease activity 3) the consideration of subgroups based on prognostic factors and potential treatment effect modifiers 4) the incorporation of realistic treatment patterns based on clinical practice/registry datasets 5) incorporation of non-joint related (extra-articular) outcomes and 6) mortality based on disease activity.

Implementation of these structural changes could be prioritized based on the expected impact on model estimates and on availability of data to populate the model. Incorporation of subgroups based on prognostic factors ranked high on the prioritization order as this is relatively straightforward. In addition, recent cost effectiveness analyses have demonstrated the importance of incorporation of subgroups as these patients may have characteristics which are potentially effect modifiers [29,30]. However, these analyses are still limited and further work needs to be done to understand and define RA subgroups with combinations of prognostic factors. Stratification of cost-effectiveness analysis by subgroups could have implication for targeting specific therapies or combination of therapies to certain subgroups thereby improving the overall clinical outcomes and cost. This could pave the way for policies leading to personalized medicine in RA.

The second priority is deemed to be the use of a disease activity measure, to model treatment response/stopping, disease progression, mapping of utility and mortality. The conceptual model allows for at least two disease activity measures one as base case and one as sensitivity analysis. Until an objective measure of disease activity is established in RA and used in routine clinical practice, impact of treatment on multiple disease activity measures will have to be evaluated in the same model/analysis. Though current mapping algorithms for utility use mixed models based on HAQ, pain and disease activity. We believe further research is required comparing mapping algorithms using different disease activity measures. In addition, future research should also evaluate the benefits of having direct measurement of utility from RA clinical trials or from real world studies vs. mapping EQ-5D.

Next on the priority list is the incorporation of more realistic treatment algorithms into the cost effectiveness model. Components of this proposed change such as glucocorticoids and NSAID intensification, treatment discontinuation, dose escalation can be informed by current RA registries, administrative claims and EMR database analysis. However, bDMARD dose de-escalation is a new development based on a recent de-escalation trial design [37–40]. Current evidence on real world dose de-escalation will be limited and hence the model will have to be informed by clinical trial data at present. The last prioritized item is the incorporation of extra-articular manifestation since more research is needed to develop RA-specific risk models for both CV and pulmonary disease however, in the interim, treatment-specific risk reduction of CV could be incorporated in sensitivity analysis.

The conceptual model presented in this manuscript concurs with some of the recommendations of the consensus recommendations from the 2015 ‘Consensus Working Party’ such as incorporation of AE based discontinuation, mapping of utility to disease activity [95]. However, there are also some major differences between the Consensus Working Party’s recommendation and the current proposed conceptual model. The reliance by the Consensus Working Party on DAS28 for treatment response could lead to biased estimates for therapies such as anti-IL6 that have a disproportionate impact on acute phase reactants in DAS28. Additional differences include incorporation of detailed treatment patterns versus only treatment discontinuation, specification of prognostic factors and incorporation of extra-articular manifestations.

This analysis represent the first step in a model building exercise, the appropriate next step would be to build a model prototype and evaluate the feasibility of operationalizing the proposed changes. Limitations of the current approach is that the analysis informing the conceptual model was based on data from one US registry. Additional work based on other registries, especially non-US registries, early RA registries and even registries focused on “treat to target” approaches would be informative. Additional limitations, include the scope of the conceptual model did not permit empirical evaluation of the proposed changes in reducing the current uncertainty in economic models and the reliance on EQ-5D as measure of utility. Finally, we did not evaluate the association between CDAI reduction and resource use/cost. However, there is evidence that attainment of remission and LDA is associated with lower resource utilization, higher quality of life and improved physical functioning [45].

Though we developed this conceptual model to meet payer/HTA needs, the focus has been on payers using cost per QALY or cost per life year gained as a metric for decision-making. Thus, our proposed model might not be applicable to address certain payer needs. For example US payers are interested in shorter time horizon with outcomes such as remission. In addition, treatment modules will have to be adapted to each country based on clinical practice data. Due to our focus on HTA bodies that consider only direct cost in economic evaluations, our model does not accommodate the indirect cost of RA into the analysis. Further work is required to specifically address modeling of cost effectiveness from the societal perspective. Finally, it was beyond the scope of this analysis to evaluate the impact of the availability of robust, comparative, head-to-head clinical trials in reducing the short-term efficacy uncertainties in economic evaluations of RA products.

Despite these limitation the conceptual model presented in this manuscript is based on a comprehensive approach that aims to incorporate both clinical and real-world evidence in the economic evaluation of RA interventions. We believe that the proposed model framework can potentially serve as a foundation for developing future cost effectiveness models in RA.

## Supporting information

S1 Appendix Working Groups RA CEA model framework “Wish List”. (DOCX)

S2 Appendix RA draft cost effectiveness model concept. (DOCX)

S3 Appendix Systematic literature review results. (DOCX)

S4 Appendix Disease activity (change) overtime and association of HAQ change by baseline DAS categories. (DOCX)

## References

[1] Scott DL, Wolfe F, Huizinga TW. Rheumatoid arthritis. Lancet 2010;376:1094–108. 10.1016/S0140-6736(10)60826-4 20870100

[2] Silman AJ, Hochberg MC. Epidemiology of the rheumatic diseases. 2nd edition: Oxford University Press; 2001.

[3] Sacks JJ, Luo YH, Helmick CG. Prevalence of specific types of arthritis and other rheumatic conditions in the ambulatory health care system in the United States, 2001–2005. Arthritis Care Res. 2010;62(4):460–464 10.1002/acr.20041 20391499

[4] van den Hoek J, Boshuizen HC, Roorda LD, Tijhuis GJ, Nurmohamed MT, van den Bos GAM, et al Mortality in patients with rheumatoid arthritis: a 15-year prospective cohort study. See comment in PubMed Commons below Rheumatol Int. 2017;37(4):487–49. 10.1007/s00296-016-3638-5 28032180 PMC5357293

[5] Uhlig T, Loge JH, Kristiansen IS. Quantification of reduced health-related quality of life in patients with rheumatoid arthritis compared to the general population. J Rheumatol. 2007;34(6):1241–7. 17516624

[6] Geryk LL, Carpenter DM, Blalock SJ, DeVellis RF, Jordan JM. The impact of co-morbidity on health-related quality of life in rheumatoid arthritis and osteoarthritis patients. Clin Exp Rheumatol. 2015;33(3):366–74. 25898121 PMC8985847

[7] Nikiphorou E, Guh D, Bansback N. Work disability rates in RA. Results from an inception cohort with 24 years follow-up. Rheumatology (Oxford). 2012;51(2):385–92. 22237045 10.1093/rheumatology/ker401

[8] Michaud K, Messer J, Choi HK, Wolfe F. Direct medical costs and their predictors in patients with rheumatoid arthritis: a three-year study of 7,527 patients. Arthritis Rheum. 2003;48(10):2750–62. 10.1002/art.11439 14558079

[9] Mennini FS, Marcellusi A, Gitto L, Iannone F. Economic Burden of Rheumatoid Arthritis in Italy: Possible Consequences on Anti-Citrullinated Protein Antibody-Positive PatientsSee comment in PubMed Commons below. Clin Drug Investig. 2017;37(4):375–386. 10.1007/s40261-016-0491-y 28074337

[10] Kirchhoff T, Ruof J, Mittendorf T, Rihl M, Bernateck M, Zeidler H, et al Cost of illness in rheumatoid arthritis in Germany in 1997–98 and 2002: cost drivers and cost savings. Rheumatology (Oxford). 2011;50(4):756–61. 21149243 10.1093/rheumatology/keq398

[11] Nam JL, et al Efficacy of biological disease-modifying antirheumatic drugs: a systematic literature review informing the 2016 update of the EULAR recommendations for the management of rheumatoid arthritis. Ann Rheum Dis. 2017 6;76(6):1113–1136. 10.1136/annrheumdis-2016-210713 28283512

[12] Feldmann M, Maini RN. Perspectives From Masters in Rheumatology and Autoimmunity: Can We Get Closer to a Cure for Rheumatoid Arthritis? Arthritis Rheumatol. 2015 9;67(9):2283–91. 10.1002/art.39269 26138641

[13] Taylor PC, Williams RO. Combination cytokine blockade: the way forward in therapy for rheumatoid arthritis? Arthritis Rheumatol. 2015 1;67(1):14–6. 10.1002/art.38893 25302944

[14] Kobelt G, Eberhardt K, Jönsson L, Jönsson B. Economic consequences ofthe progression of rheumatoid arthritis in Sweden. Arthritis Rheum 1999, 42(2):347–356. 10.1002/1529-0131(199902)42:2<347::AID-ANR18>3.0.CO;2-P 10025930

[15] Kobelt G, Jönsson L, Young A, Eberhardt K. The cost-effectiveness of infliximab (Remicade) in the treatment of rheumatoid arthritis in Sweden and the United Kingdom based on the ATTRACT study. Rheumatology (Oxford). 2003 2;42(2):326–35 12595631 10.1093/rheumatology/keg107

[16] Putrik P, Ramiro S, Kvien TK, Sokka T, Pavlova M, Uhlig T, et al Inequities in access to biologic and synthetic DMARDs across 46 European countries. Ann RheumDis 2014;73:198–206. 10.1136/annrheumdis-2012-202603 23467636

[17] Jonsson B, Kobelt G, Smolen J. The burden of rheumatoid arthritis and access to treatment: uptake of new therapies. Eur J Health Econ (2008) 8 (Suppl 2):S61–S86 18097697 10.1007/s10198-007-0089-7

[18] Van Gestel AM, Prevoo MLL, Van ‘t Hof MA, et al van Rijswijk MH, van de Putte LBA, van Riel PLCM. Development and validation of the European league against rheumatism response criteria for rheumatoid arthritis. Arthritis Rheum 1996;39(1):34–40. 8546736 10.1002/art.1780390105

[19] American College of Rheumatology Committee to Reevaluate Improvement Criteria. A proposed revision to the ACR20: the hybrid measure of American college of rheumatology. Arthritis Rheum 2007;57(2):193–202. 10.1002/art.22552 17330293

[20] Prevoo MLL, Van’t Hof MA, Kuper HH, van Leeuwen MA, van de Putte LB, van Riel PL. Modified Disease Activity Scores that include twenty-eight-joint counts. Development and validation in a prospective longitudinal study of patients with Rheumatoid Arthritis. Arthritis Rheum 1995;38(1):44–8. 7818570 10.1002/art.1780380107

[21] Smolen JS, Landewe R, Bijlsma J, Dougados M, Emery P, Gaujoux-Viala C, et al EULAR recommendations for the management of rheumatoid arthritis with synthetic and biological disease-modifying antirheumatic drugs: 2016 update. Ann Rheum Dis 2017;0:1–18. 10.1136/annrheumdis-2016-210715 28264816

[22] Singh JA, Furst DE, Bharat A, Curtis JR, Kavanaugh AF, Kremer JM, et al 2012 update of the 2008 American College of Rheumatology recommendations for the use of disease-modifying antirheumatic drugs and biologic agents in the treatment of rheumatoid arthritis. Arthritis Care Res. 2012;64(5):625–39. 10.1002/acr.21641 PMC4081542 22473917

[23] Schoels M, Alasti F, Smolen JS, Aletaha D. Evaluation of newly proposed remission cut-points for disease activity score in 28 joints (DAS28) in rheumatoid arthritis patients upon IL-6 pathway inhibition. Arthritis Res Ther. 2017 7 4;19(1):155 10.1186/s13075-017-1346-5 28676129 PMC5496440

[24] Bruce B, Fries JF: The Stanford health assessment questionnaire (HAQ): a review of its history, issues, progress, and documentation. *J Rheumatol* 2003;30 **(**1):167–78. 12508408

[25] Gibson L, Hernandez Alava M, Wailoo A. Progression of disease in people with rheumatoid arthritis treated with non biologic therapies.[Internet. Accessed Jan 12, 2018.] http://scharr.dept.shef.ac.uk/nicedsu/wp-content/uploads/sites/7/2016/03/RA-HAQ-progression-FINAL-sent-to-NICE-06.02.15-updated-12.02.15.pdf

[26] Hernández Alava M, Wailoo A, Wolfe F, Michaud K. The relationship between EQ-5D, HAQ and pain in patients with rheumatoid arthritis. Rheumatology (Oxford). 2013;52(5):944–50 23339232 10.1093/rheumatology/kes400 PMC3630395

[27] EuroQol Group. EuroQol—a new facility for the measurement of health-related quality of life. Health Policy. 1990 12;16(3):199–208. 10109801 10.1016/0168-8510(90)90421-9

[28] Tran-Duy A, Boonen A, Kievit W, van Riel PLMC, van de Laar MAFJ, Severens JL. Modelling outcomes of complex treatment strategies following a clinical guideline for treatment decisions in patients with rheumatoid arthritis. Pharmacoeconomics. 2014 10;32(10):1015–28 10.1007/s40273-014-0184-4 24972589

[29] Stevenson MD, Wailoo AJ, Tosh JC, Hernandez-Alava M, Gibson LA, Stevens JW, et al The Cost-effectiveness of Sequences of Biological Disease-modifying Antirheumatic Drug Treatment in England for Patients with Rheumatoid Arthritis Who Can Tolerate Methotrexate. J Rheumatol. 2017;44(7):973–980. 10.3899/jrheum.160941 28202743

[30] Alemao E, Johal S, Al MJ, Rutten-van Molken MPMH. Cost-Effectiveness Analysis of Abatacept Compared with Adalimumab on Background Methotrexate in Biologic-Naive Adult Patients with Rheumatoid Arthritis and Poor Prognosis. Value in Health 2018;21(2):193–202 10.1016/j.jval.2017.05.020 29477401

[31] Hochberg MC, Johnston SS, John AK. The incidence and prevalence of extra-articular and systemic manifestations in a cohort of newly-diagnosed patients with rheumatoid arthritis between 1999 and 2006. Curr Med Res Opin. 2008;24(2):469–80 10.1185/030079908X261177 18179735

[32] Prete M, Racanelli V, Digiglio L, Vacca A, Dammacco F, Perosa F. Extra-articular manifestations of rheumatoid arthritis: An update. Autoimmun Rev. 2011 12;11(2):123–31 10.1016/j.autrev.2011.09.001 21939785

[33] Prete M, Racanelli V, Digiglio L, Vacca A, Dammacco F, Perosa F. Extra-articular manifestations of rheumatoid arthritis: An update. Autoimmun Rev. 2011 12;11(2):123–31 10.1016/j.autrev.2011.09.001 21939785

[34] Kelly CA, Saravanan V, Nisar M, Arthanari S, Woodhead FA, Price-Forbes AN, et al Rheumatoid arthritis-related interstitial lung disease: associations, prognostic factors and physiological and radiological characteristics—a large multicentre UK study. Rheumatology (Oxford). 2014 9;53(9):1676–82. 24758887 10.1093/rheumatology/keu165

[35] Turesson C, McC lelland RL, Christianson TJ, Matteson ML. Multiple extra-articular manifestations are associated with poor survival in patients with rheumatoid arthritis. Ann Rheum Dis. 2006 65(11): 1533–1534. 10.1136/ard.2006.052803 17038457 PMC1798335

[36] Kang EH, Jin YP, Brill, G, Lewey JP, Patorno EP, Desai R, et al. Comparative cardiovascular safety of abatacept and tumor necrosis factor inhibitors in rheumatoid arthritis patients with and without type 2 diabetes: a population-based cohort study. 10.1136/annrheumdis-2017-eular.2407 PMC5850244 29367417

[37] Barnabe C, Martin BJ, Ghali WA. Systematic review and meta-analysis: anti-tumor necrosis factor alpha therapy and cardiovascular events in rheumatoid arthritis. Arthritis Care Res (Hoboken). 2011;63:522–529 20957658 10.1002/acr.20371

[38] Saraux A, Gossec L, Goupille P, Bergman B, Boccard E, Dupont D, et al Cost-effectiveness modelling of biological treatment sequences in moderate to severe rheumatoid arthritis in France. J Rheum 2010;49(4):733–740. 10.1093/rheumatology/kep434 20081224

[39] Ghiti Moghadam M, Vonkeman HE, Ten Klooster PM, Tekstra J, van Schaardenburg D, Starmans-Kool M, et al Stopping Tumor Necrosis Factor Inhibitor Treatment in Patients With Established Rheumatoid Arthritis in Remission or With Stable Low Disease Activity: A Pragmatic Multicenter, Open-Label Randomized Controlled Trial. Arthritis Rheumatol. 2016 8;68(8):1810–7. 10.1002/art.39626 26866428

[40] Verhoef LM, Tweehuysen L, Hulscher ME, Fautrel B, den Broeder AA. bDMARD Dose Reduction in Rheumatoid Arthritis: A Narrative Review with Systematic Literature Search. Rheumatol Ther. 2017;4(1):1–24. 10.1007/s40744-017-0055-5 28255897 PMC5443724

[41] Schett G, Emery P, Tanaka Y, Burmester G, Pisetsky DS, Naredo E, et al Tapering biologic and conventional DMARD therapy in rheumatoid arthritis: current evidence and future directions. See comment in PubMed Commons below Ann Rheum Dis. 2016;75(8):1428–39 10.1136/annrheumdis-2016-209201 27261493

[42] Emery P, Burmester GR, Bykerk VP, Combe BG, Furst DE, Barré E, et al Evaluating drug-free remission with abatacept in early rheumatoid arthritis: results from the phase 3b, multicentre, randomised, active-controlled AVERT study of 24 months, with a 12-month, double-blind treatment period. Ann Rheum Dis. 2015;74(1):19–26. 10.1136/annrheumdis-2014-206106 25367713 PMC4283672

[43] Smolen JS, Nash P, Durez P, Hall S, Ilivanova E, Irazoque-Palazuelos F, et al Maintenance, reduction, or withdrawal of etanercept after treatment with etanercept and methotrexate in patients with moderate rheumatoid arthritis (PRESERVE): a randomised controlled trial. Lancet.2013;381(9870):918–29. 10.1016/S0140-6736(12)61811-X 23332236

[44] Roberts M, Russell LB, Paltiel AD, Chambers M, McEwan P, Krahn M. Conceptualizing a model: a report of the ISPOR-SMDM modeling good research practices task force–2. Med Decis Mak 2012;32:678–89. 10.1177/0272989X12454941 22990083

[45] Alemao E, Joo S, Kawabata H, Al MJ, Allison PD, Rutten-van Molken MPMH, et al Effects of Achieving Target Measures in Rheumatoid Arthritis on Functional Status, Quality of Life, and Resource Utilization: Analysis of Clinical Practice Data. Arthritis Care Res (Hoboken). 2016;68(3):308–17. 26238974 10.1002/acr.22678 PMC5067571

[46] Alemao E, Guo Z, Frits ML, Iannaccone CK, Shadick NA, Weinblatt ME. Association of Anti-Cyclic Citrullinated Protein Antibodies, Erosions, and Rheumatoid Factor with Disease Activity and Work Productivity: A Patient Registry Study. Seminars in Arthritis & Rheumatism 2018;47(5):630–638. 29241640 10.1016/j.semarthrit.2017.10.009

[47] Alemao E, Cawston H, Bourhis F, Al MJ, Rutten-van Molken MPMH, Liao KP, et al Comparison of cardiovascular risk algorithms in patients with vs without rheumatoid arthritis and the role of C-reactive protein in predicting cardiovascular outcomes in rheumatoid arthritis. Rheumatology. 2017 5 1;56(5):777–786 10.1093/rheumatology/kew440 28087832 PMC8344293

[48] Alemao E, Cawston H, Bourhis F, Al MJ, Rutten-van Molken MPMH, Liao KP, et al Cardiovascular risk factor management in patients with RA compared to matched non-RA patients. Rheumatology (Oxford). 2016 5;55(5):809–16. 26705329 10.1093/rheumatology/kev427 PMC4830910

[49] Scholz S, Mittendorf T. Modeling rheumatoid arthritis using different techniques—a review of model construction and results. Health Econ Rev See comment in PubMed Commons below 2014 12;4(1):18 10.1186/s13561-014-0018-2 26208921 PMC4502067

[50] http://www.brassstudy.org.

[51] Aletaha D, Smolen JS. The Simplified Disease Activity Index (SDAI) and Clinical Disease Activity Index (CDAI) to monitor patients in standard clinical care. Best Practice & Research Clinical Rheumatology 2007; 21(4):663–675 17678828 10.1016/j.berh.2007.02.004

[52] Allison PD. Fixed Effects Regression Methods for Longitudinal Data Using SAS 2005

[53] Kievit W, van Herwaarden N, van den Hoogen FH, van Vollenhoven RF, Bijlsma JWJ, van den Bemt BJF, et al Disease activity-guided dose optimization of adalimumab and etanercept is a cost-effective strategy compared with non-tapering tight control rheumatoid arthritis care: analyses of the DRESS study. Ann Rheum Dis. 2016;75(11):1939–1944. 10.1136/annrheumdis-2015-208317 26764260

[54] Vermeer M, Kievit W, Kuper HH, Braakman-Jansen LMA, Bernelot Moens HJ, Zijlstra TR, et al Treating to the target of remission in early rheumatoid arthritis is cost-effective: results of the DREAM registry. BMC Musculoskelet Disord. 2013;14:350 10.1186/1471-2474-14-350 24330489 PMC3884120

[55] Eriksson JK, Karlsson JA, Bratt J, Petersson IF, van Vollenhoven RF, Ernestam S, et al Cost-effectiveness of infliximab versus conventional combination treatment in methotrexate-refractory early rheumatoid arthritis: 2-year results of the register-enriched randomised controlled SWEFOT trial. Ann Rheum Dis. 2015;74(6):1094–101. 10.1136/annrheumdis-2013-205060 24737786 PMC4431324

[56] Manders SHM, Kievit W, Adang E, Brus HL, Bernelot Moens HJ, Hartkamp A, et al Cost-effectiveness of abatacept, rituximab, and TNFi treatment after previous failure with TNFi treatment in rheumatoid arthritis: a pragmatic multi-centre randomised trial. Arthritis Res Ther. 2015;17:134 10.1186/s13075-015-0630-5 25997746 PMC4489004

[57] de Jong PH, Hazes JM, Buisman LR, Barendregt PJ, van Zeben D, van der Lubbe PA, et al Best cost-effectiveness and worker productivity with initial triple DMARD therapy compared with methotrexate monotherapy in early rheumatoid arthritis: cost-utility analysis of the tREACH trial. Rheumatology (Oxford). 2016;55(12):2138–2147. 27581208 10.1093/rheumatology/kew321

[58] Davies A, Cifaldi MA, Segurado OG, Weisman MH. Cost-effectiveness of sequential therapy with tumor necrosis factor antagonists in early rheumatoid arthritis. J of Rheum 2009:36(1);16–26. 19012363 10.3899/jrheum.080257

[59] Finckh A, Bansback N, Marra CA, Anis AH, Michaud K, Lubin S, et al Treatment of very early rheumatoid arthritis with symptomatic therapy, disease-modifying antirheumatic drugs, or biologic agents: a cost-effectiveness analysis. Annals of Internal Medicine,2009:151(9);612–621. 10.7326/0003-4819-151-9-200911030-00006 19884622

[60] Kobelt G, Lindgren P, Geborek P. Costs and outcomes for patients with rheumatoid arthritis treated with biological drugs in Sweden: A model based on registry data. Scandinavian Journal of Rheumatology 2009:38(6);409–418. 10.3109/03009740902865464 19922015

[61] Schipper LG, Kievit W, den Broeder AA, van der Laar M, Adang EMM, Fransen J, et al Treatment strategies aiming at remission in early rheumatoid arthritis patients: starting with methotrexate monotherapy is cost-effective. Rheumatology 2011:50(7):1320–1330. 10.1093/rheumatology/ker084 21371999

[62] Spalding J, Hay J. Cost-effectiveness of tumour necrosis factor-alpha inhibitors as first-line agents in rheumatoid arthritis. Pharmacoeconomics 2006, 24(12):1221–1232. 17129076 10.2165/00019053-200624120-00006

[63] Tanno M, Nakamura I, Ito K, Tanaka H, Ohta H, Kobayashi M, et al Modelling and cost-effectiveness analysis of etanercept in adults with rheumatoid arthritis in Japan: a preliminary analysis. Modern Rheumatology 2006:16(2);77–84 10.1007/s10165-006-0461-y 16633926

[64] Brennan A, Bansback N, Nixon R, Madan J, Harrison M, Watson K, et al Modelling the cost effectiveness of TNF-alpha antagonists in the management of rheumatoid arthritis: results from the British Society of Rheumatology Biologics Registry. Rheumatology 2007, 46:1345–1354. 10.1093/rheumatology/kem115 17562686

[65] Kielhorn A, Porter D. Diamantopoulos A, Lewis G. UK cost-utility analysis of rituximab in patients with rheumatoid arthritis that failed to respond adequately to a biologic disease-modifying antirheumatic drug. Curr Med Res Opin. 2008:24(9);2639–2650 10.1185/03007990802321683 18687164

[66] Vera-Llonch M, Massarotti E, Wolfe F, Shadick N, Westhovens R, Sofrygin O, et al Cost-effectiveness of abatacept in patients with moderately to severly active rheumatoid arthritis and inadequate response to methotrexate. J Rheum 2008;35(9):1745–53 18634164

[67] Wailoo AJ, Bansback N, Brennan A, Michaud K, Nixon RM, Wolfe F. Biologic drugs for rheumatoid arthritis in the medicare program: A costeffectiveness analysis. Arthritis Rheum 2008, 58(4):939–946. 10.1002/art.23374 18383356

[68] Russell A, Beresniak A, Bessette L, Haraoui B, Rahman P, Thorne C, et al Cost-effectiveness modeling of abatacept versus other biologic agents in DMARDs and anti-TNF inadequate responders for the management of moderate to severe rheumatoid arthritis. Clin Rheumatol 2009, 28(4):403–412. 10.1007/s10067-008-1060-4 19089488

[69] Hallinen TA, Soini EJO, Eklund K, Puolakka K. Cost–utility of different treatment strategies after the failure of tumour necrosis factor inhibitor in rheumatoid arthritis in the Finnish setting. J Rheum 2010, 49(4):767–777. 10.1093/rheumatology/kep425 PMC2838414 20100793

[70] Lekander I, Borgstrom F, Svarvar P, Ljung T, Carli C, van Vollenhoven RF. Cost effectiveness of real-world infliximab use in patients with rheumatoid arthritis in Sweden. Int J Technol Assess Health Care 2010;26(1):54–61. 10.1017/S0266462309990596 20059781

[71] Merkesdal S, Kirchhoff T, Wolka D, Ladinek G, Kielhorn A, Rubbert-Roth A. Cost-effectiveness analysis of rituximab treatment in patients in Germany with rheumatoid arthritis after etanercept-failure. Eur J Health Econ 2010;11(1):95–104. 10.1007/s10198-009-0205-y 19967426

[72] Yuan Y, Trivedi D, Maclean R, Rosenblatt L. Indirect cost-effectiveness analyses of abatacept and rituximab in patients with moderate-to-severe rheumatoid arthritis in the United States. J Med Econ 2010, 13(1):33–41. 10.3111/13696990903508021 20001596

[73] Kobelt G, Lekander I, Lang A, Raffeiner B, Botsios C, Geborek P. Cost effectiveness of etanercept treatment in early active rheumatoid arthritis followed by dose adjustment. Int J Technol Assess Health Care 2011;27(03):193–200. 21736857 10.1017/S0266462311000195

[74] Valle-Mercado C, Cubides MF, Parra-Torrado M, Rosselli D. Cost-effectiveness of biological therapy compared with methotrexate in the treatment for rheumatoid arthritis in Colombia. Rheumatol Int. 2013;33(12):2993–7 10.1007/s00296-013-2834-9 23907586

[75] Tanaka E, Inoue E, Hoshi D, Shimizu Y, Kobayashi A, Sugimoto N, et al Cost-effectiveness of tocilizumab, a humanized anti-interleukin-6 receptor monoclonal antibody, versus methotrexate in patients with rheumatoid arthritis using real-world data from the IORRA observational cohort study. Mod Rheumatol. 2015;25(4):503–13. 10.3109/14397595.2014.1001475 25547018

[76] Stephens S, Botteman MF, Cifaldi MA, van Hout B. Modelling the cost-effectiveness of combination therapy for early, rapidly progressing rheumatoid arthritis by simulating the reversible and irreversible effects of the disease. BMJ Open. 2015;5(6):e006560 10.1136/bmjopen-2014-006560 26059521 PMC4466612

[77] Gissel C, Götz G, Repp H. Cost-effectiveness of adalimumab for rheumatoid arthritis in Germany. Z Rheumatol. 2016;75(10):1006–1015. 10.1007/s00393-016-0071-9 27080399 PMC5127857

[78] Ollendorf DA, Klingman D, Hazard E, Ray S. Differences in annual medication costs and rates of dosage increase between tumor necrosis factor-antagonist therapies for rheumatoid arthritis in a managed care population. Clin Ther. 2009; 31(4): 825–835. 10.1016/j.clinthera.2009.04.002 19446156

[79] Ollendorf DA, Massarotti E, Birbara C, Misra Burgess S. Frequency, predictors, and economic impact of upward dose adjustment of infliximab in managed care patients with rheumatoid arthritis. J Manag Care Pharm. 2005;11(5): 383–393. doi: 10.18553/jmcp.2005.11.5.383 15934797 10.18553/jmcp.2005.11.5.383 PMC10438098

[80] Klarenbeek NB, Koevoets R, van der Heijde DM, Gerards AH, ten Wolde S, Kerstens PJSM, et al Association with joint damage and physical functioning of nine composite indices and the 2011 ACR/EULAR remission criteria in rheumatoid arthritis. Ann Rheum Dis. 2011;70(10):1815–21. 10.1136/ard.2010.149260 21813548

[81] Gaujoux-Viala C, Mouterde G, Baillet A, Claudepierre P, Fautrel B, Le-Loet X, et al Evaluating disease activity in rheumatoid arthritis: which composite index is best? A systematic literature analysis of studies comparing the psychometric properties of the DAS, DAS28, SDAI and CDAI. Joint Bone Spine.2012;79(2):149–55. 10.1016/j.jbspin.2011.04.008 21680221

[82] Felson DT, Smolen JS, Wells G. American College of Rheumatology/European League against Rheumatism Preliminary Definition of Remission in Rheumatoid Arthritis for Clinical Trials. Arthritis Rheum. 2011; 63(3): 573–586 10.1002/art.30129 21294106 PMC3115717

[83] Vastesaeger N, Xu S, Aletaha D, St Clair EW, Smolen JS. A pilot risk model for the prediction of rapid radiographic progression in rheumatoid arthritis. Rheumatology (Oxford) 2009;48:1114–21. 19589891 10.1093/rheumatology/kep155

[84] Visser K, Goekoop-Ruiterman YP, de Vries-Bouwstra JK, Ronday HK, Seys PEH, Kerstens PJSM, et al A matrix risk model for the prediction of rapid radiographic progression in patients with rheumatoid arthritis receiving different dynamic treatment strategies: post hoc analyses from the BeSt study. Ann Rheum Dis 2010;69:1333–7. 10.1136/ard.2009.121160 20498212

[85] Syversen SW, Gaarder PI, Goll GL, Ødegård S, Haavardsholm EA, Mowinckel P, et al High anti-cyclic citrullinated peptide levels and an algorithm of four variables predict radiographic progression in patients with rheumatoid arthritis: results from a 10-year longitudinal study. Ann Rheum Dis. 2008 7; 67(2): 212–217. 10.1136/ard.2006.068247 17526555

[86] Humphreys JH, van Nies JAB, Chipping J, Marshall T, van der Helm-van Mil AHM, Symmons DPM, et al Rheumatoid factor and anti-citrullinated protein antibody positivity, but not level, are associated with increased mortality in patients with rheumatoid arthritis: results from two large independent cohorts. Arthritis Res Ther. 2014;16(6): 483 10.1186/s13075-014-0483-3 25471696 PMC4272533

[87] Vencovský J, Machacek S, Sedova L, Kafkova J, Gatterova J, Pešákováb V, et al Autoantibodies can be prognostic markers of an erosive disease in early rheumatoid arthritis. Ann Rheum Dis. 2003;62(5):427–430 10.1136/ard.62.5.427 12695154 PMC1754544

[88] Gottenberg JE, Courvoisier DS, Hernandez MV, Iannone F, Lie E, Canhao H, et al A Brief Report: Association of Rheumatoid Factor and Anti-Citrullinated Protein Antibody Positivity With Better Effectiveness of Abatacept: Results From the Pan-European Registry Analysis. Arthritis Rheumatol. 2016;68(6):1346–52. 10.1002/art.39595 26815727

[89] Widdifield J, Bernatsky S, Paterson JM, Gunraj N, Thorne JC, Pope J, et al Serious infections in a population-based cohort of 86,039 seniors with rheumatoid arthritis. Arthritis Care Res (Hoboken). 2013 3;65(3):353–61. 22833532 10.1002/acr.21812

[90] Meissner Y, Zink A, Kekow J, Rockwitz K, Liebhaber A, Zinke S, et al Impact of disease activity and treatment of comorbidities on the risk of myocardial infarction in rheumatoid arthritis. Arthritis Res Ther. 2016 8 5;18(1):183 10.1186/s13075-016-1077-z 27495156 PMC4975917

[91] Harrold LR, Litman HJ, Connolly SE, Kelly S, Hua W, Alemao E, et al Impact of anti-cyclic citrullinated peptide and rheumatoid factor status on response to abatacept therapy: Findings from a US observational cohort Ann Rheum Dis 2016;75:505–6

[92] Kubo S, Nakayamada S, Nakano K, Hirata S, Fukuyo S, Miyagawa I, et al Comparison of the efficacies of abatacept and tocilizumab in patients with rheumatoid arthritis by propensity score matching. Ann Rheum Dis. 2016;75(7):1321–7 10.1136/annrheumdis-2015-207784 26245754

[93] Bongartz T, Sutton AJ, Sweeting MJ, Buchan I, Matteson EL, Montori V. Anti-TNF antibody therapy in rheumatoid arthritis and the risk of serious infections and malignancies: Systematic review and meta-analysis of rare harmful effects in randomized controlled trials. JAMA 2006;295(19):2275–2285. 10.1001/jama.295.19.2275 16705109

[94] Ruyssen-Witrand A, Fautrel B, Saraux A, Le-Loet X. Pham T. Infections induced by low-dose corticosterioids in rheumatoid arthritis: A systematic literature review. Joint Bone Spine May 2010(77);246–251. 10.1016/j.jbspin.2010.02.009 20451437

[95] Madan J, Ades T, Barton P, Bojke L, Choy E, Helliwell P, et al Consensus Decision Models for Biologics in Rheumatoid and Psoriatic Arthritis: Recommendations of a Multidisciplinary Working Party. Rheumatol Ther. 2015 12;2(2):113–125. 10.1007/s40744-015-0020-0 27747536 PMC4883267

