# Supplementary material for: Conceptual model for the health technology assessment of current and novel interventions in rheumatoid arthritis
Source: PLoS One. 2018 Oct 5;13(10):e0205013. doi: 10.1371/journal.pone.0205013 (PMC6173427; doi:10.1371/journal.pone.0205013)
Supplement: S1 Appendix — (DOCX) [file pone.0205013.s001.docx]

**S1 Appendix.** Working Groups RA CEA Model Framework “Wish List”

| **Model Parameters** | **Common to most current modeling approaches** | **Proposed Model** |
| --- | --- | --- |
| Technique | Discrete event simulation, Individual patient simulation, Markov cohort | TBD* |
| Baseline Patient Characteristics | Age, gender, HAQ & weight | Demographic: age, gender, duration of disease, weight  Prognostic factors: Sero positivity (RF and/or ACPA), auto immune co-morbidities  Risk of infections: age, diabetes, BMI, corticosteroid use |
| Extra-articular manifestation | None | CVD: CV related risk factors |
| Treatment effect | ACR20, DAS28, EULAR (DAS28 baseline & change from baseline) | TBD* |
| Disease progression | HAQ | TBD* |
| Utility | through HAQ | TBD* |
| Treatment patterns | Sequential switching | Real world based  Dose escalation |
| Treatment adverse effects | Same across all treatments | Treatment class specific |
|  | Same across all patients | Subgroups of patients at high risk for AEs and infections |

*** To be determined based on literature and database analysis**
