# Supplementary figures and images for: Conceptual model for the health technology assessment of current and novel interventions in rheumatoid arthritis
Source: PLoS One. 2018 Oct 5;13(10):e0205013. doi: 10.1371/journal.pone.0205013 (PMC6173427; doi:10.1371/journal.pone.0205013)

**S2 Appendix.** RA Draft Cost Effectiveness Model Concept


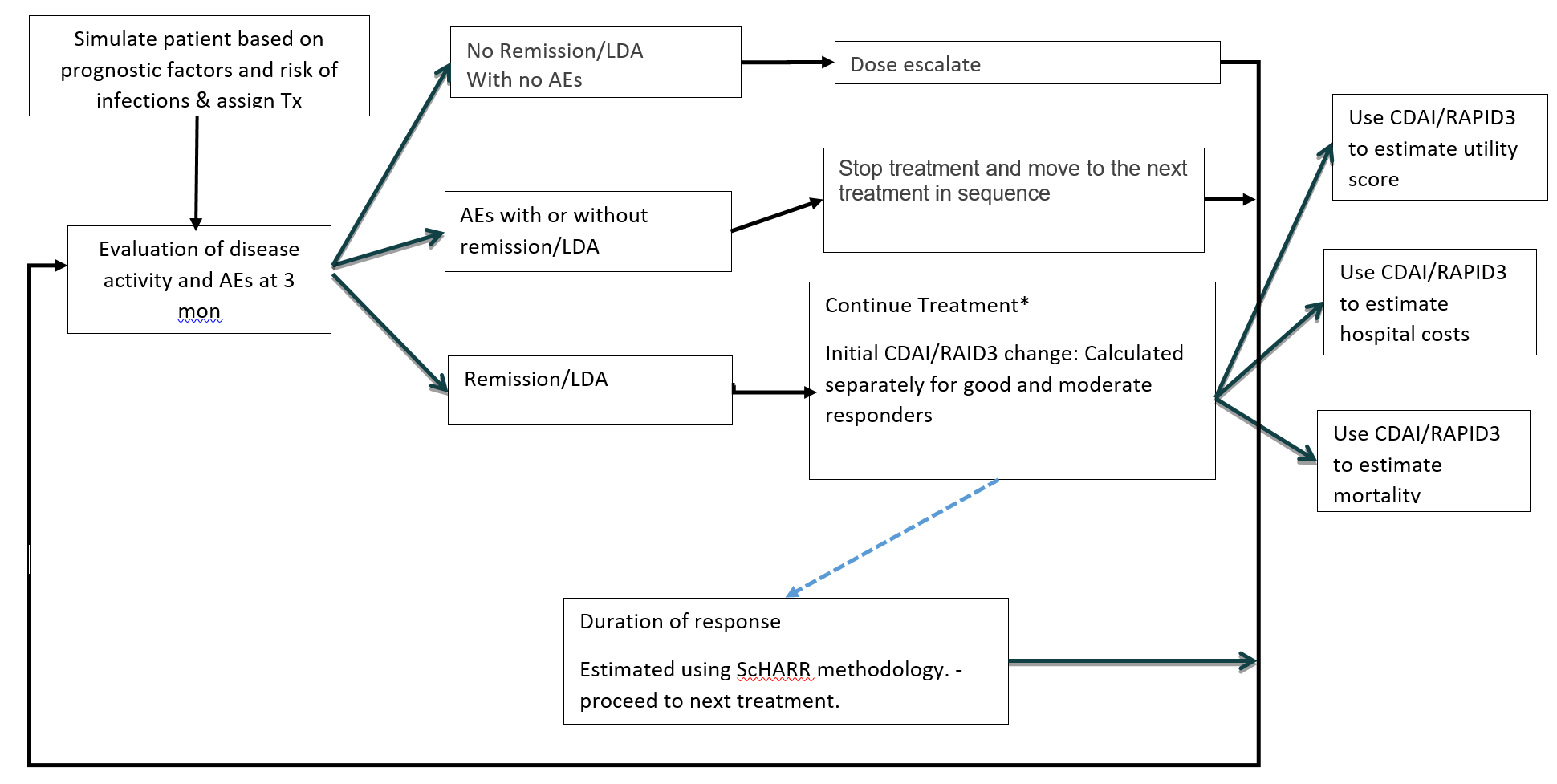

Supplement: S2 Appendix — (DOCX) [file pone.0205013.s002.docx]
