# Supplementary material for: Conceptual model for the health technology assessment of current and novel interventions in rheumatoid arthritis
Source: PLoS One. 2018 Oct 5;13(10):e0205013. doi: 10.1371/journal.pone.0205013 (PMC6173427; doi:10.1371/journal.pone.0205013)
Supplement: S3 Appendix — (DOCX) [file pone.0205013.s003.docx]

**S3 Appendix. Systematic Literature Review Results**

**S3 Table 1.** Literature Search Strategy

| **#** | **Search Strategy** |
| --- | --- |
| **1** | rheumatoid arthritis.mp. [mp=ti, ab, sh, hw, tn, ot, dm, mf, dv, kw, ps, rs, nm, ui, ct] |
| **2** | arthritis, rheumatoid/ or rheumatoid arthritis/ |
| **3** | (tocilizumab or Actemra or RoActemra or golimumab or simponi or adalimumab or humira or etanercept or enbrel or rituximab or mabthera or abatacept or orencia or infliximab or remicade or certolizumab pegol or cimzia).mp. [mp=ti, ab, sh, hw, tn, ot, dm, mf, dv, kw, ps, rs, nm, ui, ct] |
| **4** | (Anakinra or Rituxan or Methotrexate or Rhemumatrex or Trexall or Sulfasalazine or Azulfidine or Salazopyrin or Hydroxychloroquine or Plaquenil).mp. [mp=ti, ab, sh, hw, tn, ot, dm, mf, dv, kw, ps, rs, nm, ui, ct] |
| **5** | (1 or 2) and (3 or 4) |
| **6** | limit 5 to english language |
| **7** | limit 6 to human |
| **8** | limit 7 to yr="2001 -Current" |
| **9** | (economic evaluation or economic model or cost effectiveness or cost benefit or cost utility).ti,ab. |
| **10** | "cost benefit analysis"/ |
| **11** | (9 or 10) and 8 |
| **12** | (Predict$ and (Outcome$ or Risk$ or Model$)).mp. |
| **13** | (Prognostic and (History or Variable$ or Criteria or Scor$ or Characteristic$ or Finding$ or Factor$ or Model$)).mp. |
| **14** | predict*.ti. |
| **15** | (13 or 12 or 14) and 8 |
| **16** | 11 or 15 |
| **17** | remove duplicates from 16 |
| **18** | limit 17 to (editorial or letter or note or autobiography or bibliography or biography or case reports or clinical conference or clinical trial, phase i or clinical trial, phase ii or comment or in vitro or interactive tutorial or interview or lectures or legal cases or legislation or news or newspaper article or periodical index or portraits) [Limit not valid in Embase,Ovid MEDLINE(R),Ovid MEDLINE(R) In-Process,Econlit; records were retained] |
| **19** | 17 not 18 |

**S3 Figure 1.** Flowchart of study identification and selection


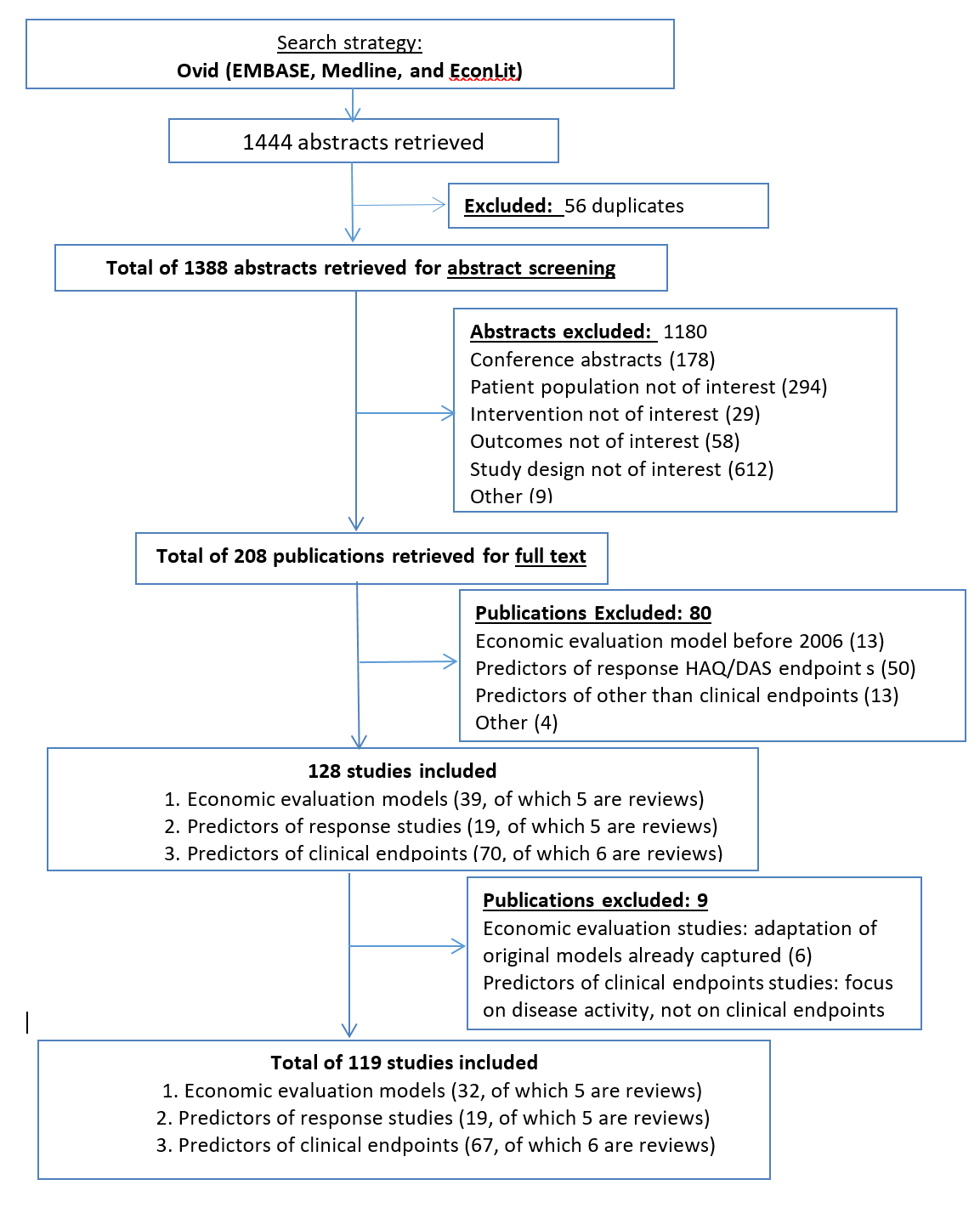


**S3 Table 2**. Summary of the Systematic Literature Review

| **Reference** | **Study Population** | **Comparators* & perspective** | **Modelling Approach** | **Initial treatment response (3 to 6 month)** | **Long-term (life time) disease progression and Outcome Modelled** | **Mortality** | **QALY** |
| --- | --- | --- | --- | --- | --- | --- | --- |
| [Kievit W](https://www.ncbi.nlm.nih.gov/pubmed/?term=Kievit%20W%5BAuthor%5D&cauthor=true&cauthor_uid=26764260) et al 2016^23^ | patients with RA using adalimumab or etanercept and enrolled in the DRESS study | - a standardized tight control treatment protocol vs. dose optimisation group, patients received identical care as the control group, with the addition of a dose reduction advice | 1000 bootstraps of 18 months mean QALY and cost outcome from DRESS trial | DAS28-CRP | none | none | Direct from EQ5D data captured in DRESS trial |
| [Vermeer M](https://www.ncbi.nlm.nih.gov/pubmed/?term=Vermeer%20M%5BAuthor%5D&cauthor=true&cauthor_uid=24330489) et al 2014^24^ | early RA patients in real-life daily clinical practice | - T2T strategy aiming at remission compared to usual care for | No model; CE based on a , 3 yr data from 2 early RA registries | DAS28 | None | None | EuroQol-5D (EQ-5D) values[[18](https://bmcmusculoskeletdisord.biomedcentral.com/articles/10.1186/1471-2474-14-350#CR18)] were estimated from the Health Assessment Questionnaire (HAQ) scores |
| [Eriksson JK](https://www.ncbi.nlm.nih.gov/pubmed/?term=Eriksson%20JK%5BAuthor%5D&cauthor=true&cauthor_uid=24737786) et al 2015^25^ | early RA patients with insufficient response to MTX | - adding infliximab or sulfasalazine and hydroxychloroquine to methotrexate in | No model; CE based on a , 21 month data from SWEFOT study | EULAR | None (21 month ICER based on trial results) | none | Direct EQ-5D from the SWEFOT trial |
| [Manders SH](https://www.ncbi.nlm.nih.gov/pubmed/?term=Manders%20SH%5BAuthor%5D&cauthor=true&cauthor_uid=25997746) et al 2015^26^ | 139 patients that failed previous TNFi treatment | - abatacept, rituximab or a different TNFi | No model; CE based on a pragmatic, 1-year randomized trial | DAS28 | None (1 year ICER based on trial results) | none | Direct EQ-5D from the pragmatic trial |
| de Jong PH et al 2016^27^ | Patients with a high probability (>70%) according to their likelihood of progressing to persistent arthritis in Rotterdam Early Arthritis Cohort | - A) initial triple DMARD therapy (iTDT) with glucocorticoids (GCs) intramuscular vs. B) iTDT with an oral GC tapering vs C) initial MTX monotherapy with GCs similar to B - societal perspective | No model; based on registry data | DAS remission | EQ5D at 1 yr | None | Directly from EQ5D |
| Davies et al. 2009^28^ | Patients with early RA in the US. | - TNFα inhibitors (infliximab, etanercept and adalimumab) + MTX as a group - Payer | - IPS model - life time horizon | ACR categorized in 4 intervals (ACR0–20, ACR20–50, ACR50–70, and ACR70–100). | - H HAQ - Cost/   QALY | Mortality (age specific all-cause mortality was adjusted using HAQ). | Function of HAQ. |
| Finckh et al. 2009^29^ | Adult very early RA (symptom duration 3 months). | - cDMARD with MTX vs bDMARDs + MTX - Healthcare provider & societal | - Individual sampling model - lifetime horizon | HAQ categorized as excellent, moderate/ good response:  induced remission using a threshold of DAS28 less than <2.6. | - HAQ - Cost/   QALY | Radiographic joint damage  Mortality (adjusted by HAQ, gender, and age). | Function of HAQ. |
| Kobelt et al. 2009^30^ | Patients with RA treated with bDMARD | - Etanercept, infliximab, adalimumab - Societal | DES model  5- and a 10-year time horizon. | HAQ and DAS | - HAQ & DAS - Cost/   QALY | Mortality (assumed to be independent of treatment lines) | Utilities were directly available in the SSATG Register dataset. |
| Schipper et al. 2011^31^ | Patients with early RA in the Netherlands. | - Three outcome-directed strategies were compared:   MTX, LEF, MTX + anti-TNF vs.  MTX+LEF combination, MTX+anti-TNF vs.  MTX + anti-TNF   - Healthcare payer and societal perspective | A Markov model with a cycle length of three months and a time horizon of 5 years. | DAS28  . | - DAS - Cost/   QALY | Not reported. | Estimated as a function of DAS28. |
| Spalding & Hay. 2006^32^ | Hypothetical cohort of women 55-60 years of age and diagnosed with RA in the US | - TNFα inhibitors (both as monotherapy and in combination with methotrexate) - Payer | - Markov model - cycle length of 1 year - lifetime time-horizon | - HAQ | - HAQ - Cost/QALY | Mortality (individualised for each of the states based on age, HAQ and gender). | Function of HAQ. |
| Tanno et al. 2006^33^ | Patients with chronic RA based on phase III clinical trial of etanercept. | - Etanercept + methotrexate (MTX) vs MTX - Societal | - Markov model - 6-month cycles - lifetime time horizon | - ACR20. | - HAQ - Cost/QALY | Mortality (individualised for each of the time cycles based on age, HAQ and gender). | Function of HAQ. |
| Brennan et al. 2007^34^ | UK NHS patients in th British Society for Rheumatology Biologics Registry with RA who have failed at least two traditional DMRDs | - TNFα inhibitors (infliximab, etanercept and adalimumab) + MTX as a group - UK NHS | - IPS model - lifetime time horizon | EULAR response criteria | - HAQ - Cost/QALY | Mortality (assumed to be equivalent in the two arms of the model). |  |
| Kielhorn et al. 2008^35^ | RA patients with inadequately to a bDMARD (patient demographics match those of patients in the pivotal rituximab trial – REFLEX). | - Rituximab + MTX vs Standard practice (leflunomide) - UK NHS and PS in England and Wales | - Micro simulation Markov model - 6-month cycles - lifetime time horizon. | ACR20, ACR50, ACR70 | - HAQ - Cost/   QALY | Mortality (individualized by HAQ score). | Function of HAQ. |
| Vera-Llonch et al. 2008^36^ | Moderate to severe RA patients with inadequate response to MTX in the US. | - Abatacept + MTX vs. MTX - Third-payer | - Simulation model - time frame of both 10 years and lifetime. | *HAQ-DI improvements of 0.50 or greater at 6 months* | - HAQ - Cost/-QALY | Mortality (individualized by age, HAQ and gender). | Function of HAQ. |
| Wailoo et al. 2008^37^ | US Medicare patients with a diagnosis of RA treated with each of the 4 bDMARDs (adalimumab, anakinra, etanercept, infliximab) | - Adalimumab, Anakinra, Etanercept, Infliximab - Medicare | - Simulation model - lifetime horizon | ACR20 and ACR50 | - HAQ - Cost/QALY | Mortality (US life tables were adjusted by standardized mortality rates for RA). | Estimated as a function of HAQ. |
| Russell et al. 2009^38^ | moderate to severe RA with an inadequate response to one or more DMARDs and/or anti-TNF agents in Canada. | 3 treatment sequences:   - Etanercept, infliximab, adalimumab, DMARDs vs. - Abatacept, etanercept, infliximab, DMARDs vs. - Etanercept, abatacept, infliximab, DMARDs - Payer | - Simulation model - Two-year time horizon. | DAS28 | - DAS - cost / additional LDAS gained. | Not relevant | Not relevant |
| Hallinen et al. 2010^39^ | Patients with severe RA after TNF-inhibitor failure in Finland. | - adalimumab vs. abatacept vs. etanercept vs. infliximab vs. - payer | - Micro-simulation Markov model - life-time horizon. | ACR (20/50/70) | - HAQ - Cost/   QALY | Mortality (adjusted by HAQ score) | function of HAQ. |
| Lekander et al. 2010^40^ | Patients with RA in Swedish clinical practice | - Infliximab vs. - natural progression (i.e., no biologic treatment) - Societal | Markov cohort model with one-yearly cycles and a 20-year time-horizon. | HAQ and DAS28 | - HAQ - Cost/   QALY | Mortality (adjusted by functional status [HAQ] and disease activity [DAS]). | Estimated as a function of HAQ. |
| Merkesdal et al. 2010^41^ | Patients with active RA who failed at least one prior DMARD therapy in Germany. | - adalimumab + MTX followed by infliximab + MTX, followed by gold, followed by cyclosporine A, and finally supportive therapy (MTX) vs. rituximab + MTX, followed by adalimumab + MTX, followed by infliximab + MTX, followed by gold, followed by cyslosporine A, and finally supportive therapy (MTX). - payer | A micro simulation Markov model with 6-month cycles and a lifetime time horizon. | ACR (20/50/70) | - HAQ - Cost/   QALY | Mortality (individualized by employing mortality tables of the German population that were adjusted according to the patient's stage of RA ). | Estimated as a function of HAQ. |
| Saraux et al. 2010^42^ | moderate to severe active RA patients and an insufficient response to at least one anti-TNF agent in France. | - 4 treatment sequences were compared:   abatacept, adalimumab vs.  rituximab, adalimumab vs.  adalimumab, abatacept vs.  adalimumab, infliximab.   - payer | Simulation-decision analytical model with a two year time-horizon | DAS28 | Not relevant | LDAS (DAS28≤3.2)  Remission (DAS28<2.6) | Estimated as a function of HAQ. |
| Yuan et al. 2010^43^ | Patients with active RA and who have had an inadequate response to anti-TNFα therapy in the US. | - Abatacept + MTX vs. Rituximab + MTX vs.MTX alone - US third-party payer | patient-level simulation model with 3-month cycles and a life time horizon. | HAQ-DI | - HAQ - Cost/   QALY | Mortality (was individualized by gender, age and HAQ). | Estimated as a function of HAQ. |
| Kobelt et al. 2011^44^ | Patients with early active RA in Sweden. | - etanercept, followed by dose-reduction in the case of remission vs.standard treatment with MTX. - Societal | Markov model with 6 monthly cycles and a 10-year time horizon. | HAQ and DAS | - HAQ & DAS - Cost/   QALY | Mortality (adjusted by HAQ and DAS28) | Estimated as a function of HAQ and DAS28. |
| [Valle-Mercado C](https://www.ncbi.nlm.nih.gov/pubmed/?term=Valle-Mercado%20C%5BAuthor%5D&cauthor=true&cauthor_uid=23907586) 2013 et al^45^ | 150 patients in different disease stages at the Hospital Militar, one of the largest university hospitals in Colombia | - BDMARDs vs. MTX | Markov model | HAQ | HAQ | standardized mortality ratio | As a function of HAQ |
| [Tanaka E](https://www.ncbi.nlm.nih.gov/pubmed/?term=Tanaka%20E%5BAuthor%5D&cauthor=true&cauthor_uid=27472516) et al 2015^46^ | RA patients failing cDMARDs enrolled in the IORRA registry | - bDMARD therapy with tociluzimab vs. without | state-transition model |  |  |  |  |
| [Stephens S](https://www.ncbi.nlm.nih.gov/pubmed/?term=Stephens%20S%5BAuthor%5D&cauthor=true&cauthor_uid=26059521) et al 2015^47^ | 1000 early RA patients based on PREMIER study | - Adalimumab + MTX versus MTX | microsimulation | ACR based on DAS28 | Disease activity and mTSS were linked to an individual’s HAQ | Not stated | derived from the HUI3, were estimated based on data from PREMIE |
| [Gissel C](https://www.ncbi.nlm.nih.gov/pubmed/?term=Gissel%20C%5BAuthor%5D&cauthor=true&cauthor_uid=27080399) et al 2016^48^ | 10,000 hypothetical RA patients based characteristics from RABBIT registry | - Adalimumab + MTX vs, MTX | individual patient sampling | ACR response | HAQ | German life tables based on age and gender | Estimated as a function of HAQ. |
| [Stevenson MD](https://www.ncbi.nlm.nih.gov/pubmed/?term=Stevenson%20MD%5BAuthor%5D&cauthor=true&cauthor_uid=28202743) et al 2017^49^ | Patients with moderate to severe RA and with severe RA with prior experience of MTX. patient characteristics from the BSRBR for those receiving their first bDMARD, | - adalimumab. etanercept. infliximab (IFX), certolizumab pegol (CTZ), golimumab (GOL), tocilizumab (TCZ), and abatacept (ABA) vs. MTX - (RTX) + MTX, then TCZ + MTX (if TCZ + MTX was not used first-line), followed by a range of nonbiologic therapies | individual patient time-to-event simulation | EULAR response | HAQ | mortality conditional on HAQ score | mixture model proposed based on HAQ & pain |

*Includes both individual treatment comparisons as well as comparison of interventions for treat to target approach
