# Supplementary material for: Conceptual model for the health technology assessment of current and novel interventions in rheumatoid arthritis
Source: PLoS One. 2018 Oct 5;13(10):e0205013. doi: 10.1371/journal.pone.0205013 (PMC6173427; doi:10.1371/journal.pone.0205013)
Supplement: S4 Appendix — (DOCX) [file pone.0205013.s004.docx]

**S4 Appendix.** Disease activity (change) overtime and association of HAQ change by baseline DAS categories

**S4 Figure 1.** CDAI, SDAI and DAS28-CRP overtime


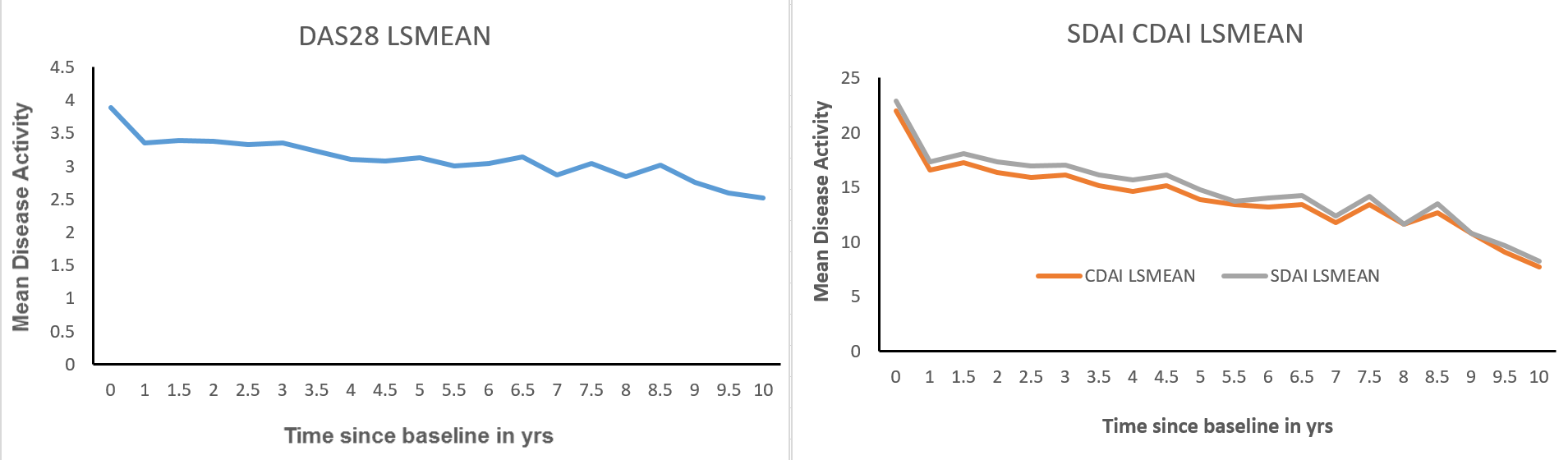


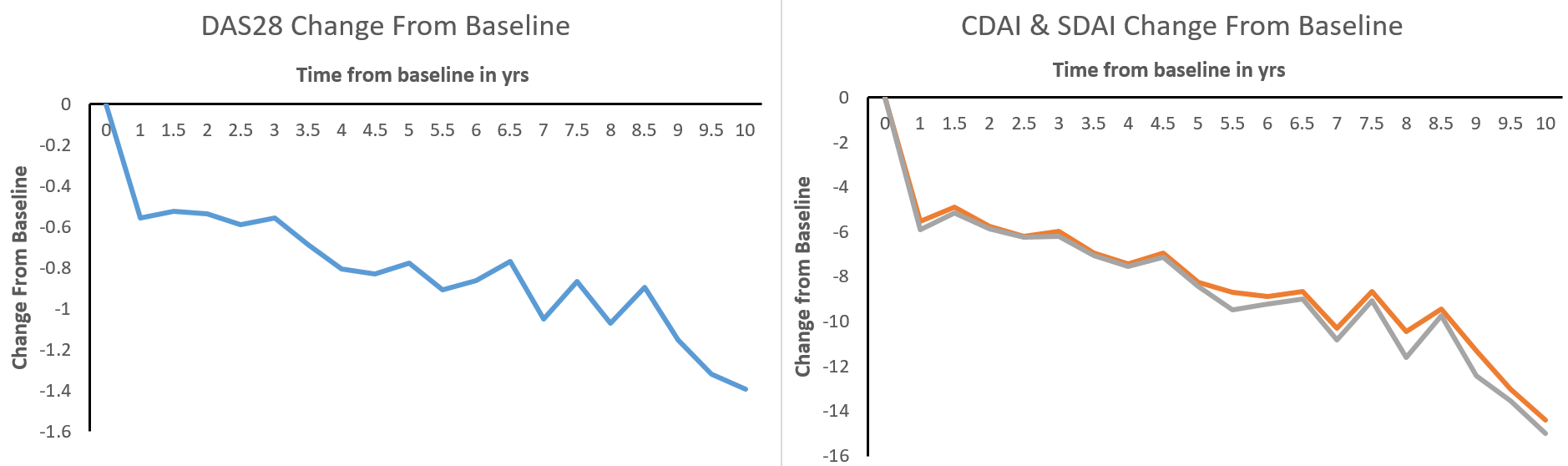


Means are based on generalized linear models adjusted for baseline age, disease activity, gender, disease duration, serostatus, CRP, time since baseline

**S4 Figure 2.** CDAI, SDAI and DAS28-CRP overtime by disease duration and change overtime


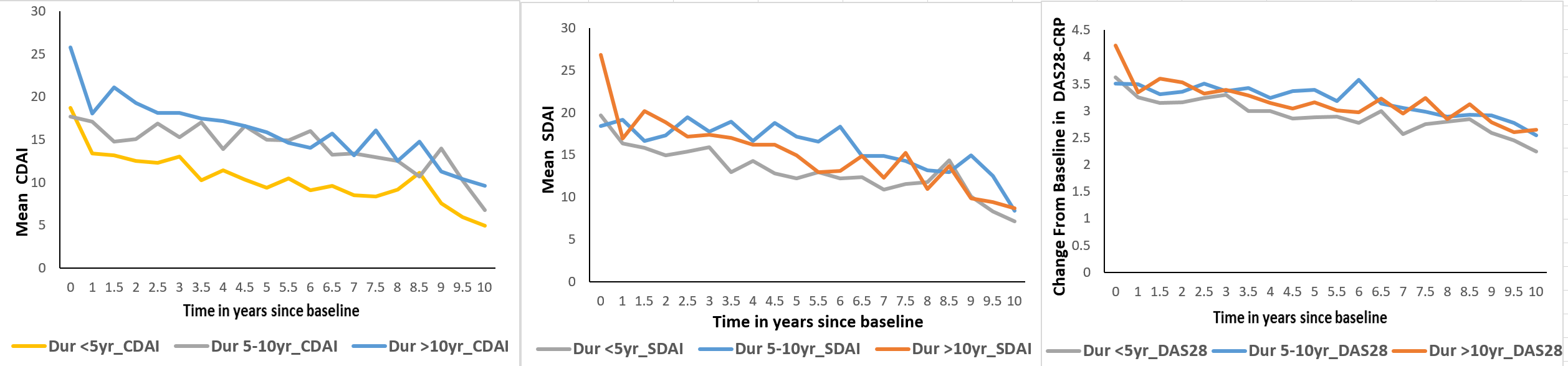


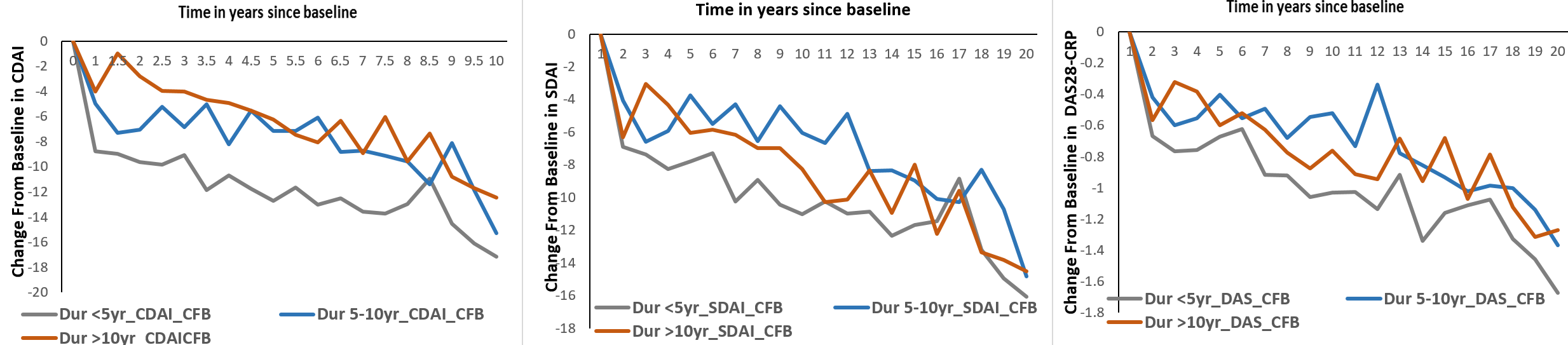


Means are based on generalized linear models adjusted for baseline age, disease activity, gender, disease duration, serostatus, CRP, time since baseline

S4 Table 1: HAQ change by DAS28 categories

|  | DAS28 =< 2.6 (remission) N = 1820 | < 2.6 DAS28 =< 3.2 (LDA) N = 608 | < 3.2 DAS28 =< 5.1 (MDA) N = 1350 | > 5.1 DAS28 =< 5.2 (SDA) N = 516 |
| --- | --- | --- | --- | --- |
|  | Mean (SE) | Mean (SE) | Mean (SE) | Mean (SE) |
| mHAQ | 0.254 (0.008) | 0.320 (0.013) | 0.399 (0.009) | 0.603(0.015) |
| mHAQ change from baseline* | 0.140 (0.008) | -0.073 (0.013) | 0.004 (0.009) | 0.209 (0.015) |

* negative change is worsening of HAQ; means based on general linear model adjusted for baseline co-variates


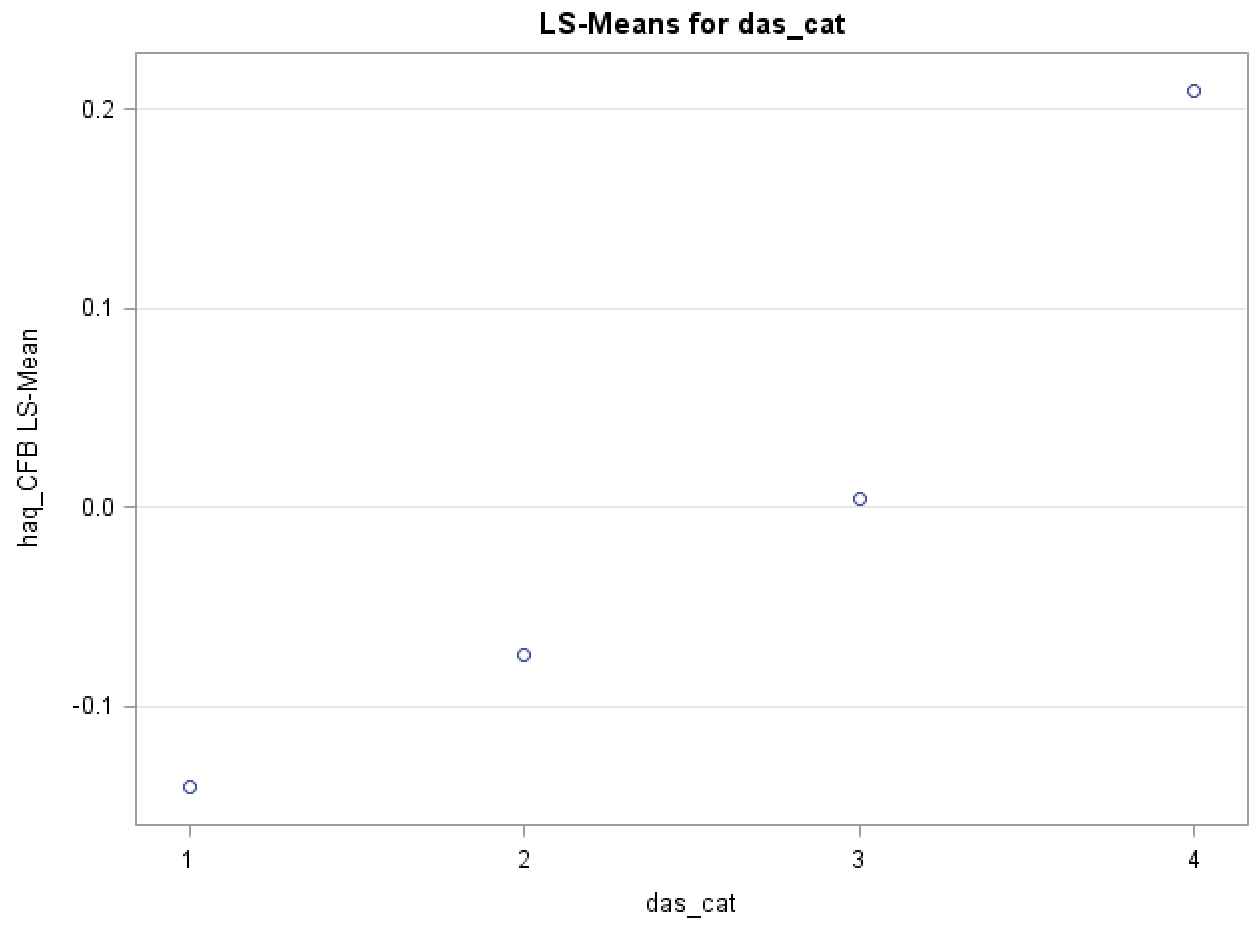


Das_cat : 1= DAS28 =< 2.6 ; 2= < 2.6 DAS28 =< 3.2 ; 3= < 3.2 DAS28 =< 5.1 ;4= > 5.1 DAS28 =< 5.2
